# Single-cell transcriptomic landscape delineates pathogenic NK cell subpopulations and key mediators in systemic inflammatory response syndrome

**DOI:** 10.1007/s10238-026-02285-2

**Published:** 2026-08-19

**Authors:** Hongling Jia, Han Zhao, Yan Li, Nuan Qian, Zhijie Zhao, Xiaojing Wang, Qi Sun, Fu Zhao, Xinyi Zhang, Lin Sun, Yuqi Zhang, Yang Hong, Haoyu Sun, Qinghan Zhang, Haiyu Li

**Affiliations:** 1 College of First Clinical Medicine, Shandong University of Traditional Chinese Medicine, Jinan, 250014 China; 2 Shanghai Jiao Tong University School of Medicine, Shanghai, China; 3 Department of Neurosurgery, The First Affiliated Hospital of Anhui Medical University, Hefei, Anhui China; 4 Henan Medical University, Xinxiang, 453000 China; 5 Department of Plastic and Reconstructive Surgery, Shanghai Ninth People’s Hospital, School of Medicine, Shanghai Jiao Tong University, Shanghai, China; 6 Department of Rheumatology and Immunology, Tongren Hospital, School of Medicine, Shanghai Jiao Tong University, Shanghai, China; 7 Shandong University of Traditional Chinese Medicine, Jinan, 250014 China; 8 School of Traditional Chinese Medicine, Jinan University, Guangzhou, China; 9 Department of Sports Medicine, Shanghai General Hospital, Shanghai Jiao Tong University School of Medicine, Shanghai Jiao Tong University, Shanghai, China; 10 Anhui Medical University, Hefei, 230032 China; 11 Department of Orthopaedic Surgery, China-Japan Union Hospital of Jilin University, Changchun, 130033 China; 12 Department of Otolaryngology, Songjiang Hospital, Shanghai Jiao Tong University School of Medicine, Shanghai, China; 13 Department of Clinical Laboratory, Songjiang Hospital, Shanghai Jiao Tong University School of Medicine, Shanghai, China

**Keywords:** Systemic Inflammatory Response Syndrome (SIRS), Single-cell RNA sequencing (scRNA-seq), Natural killer cells (NK cells), NK cell dysfunction, Innate immunity, Inflammation

## Abstract

Systemic inflammatory response syndrome (SIRS) carries high ICU morbidity, sepsis lacks standardized therapies, and stage-variable natural killer (NK) cell function in SIRS remains poorly defined due to insufficient single-cell evidence. Herein, we analyzed public LPS-induced SIRS peripheral blood single-cell RNA sequencing (scRNA-seq) data (GSE212092) via multiple bioinformatic algorithms to dissect NK heterogeneity, followed by in vitro functional verification using NK-92 cells with IGF1 stimulation or IGF1R knockdown. We identified distinct cell types and 6 distinct NK subpopulations (C0-C5). Pro-inflammatory C0 and proliferative C2 IGF1R⁺ expanded in early SIRS, whereas stemness-high C3-C5 accumulated during late immunosuppression. MHC-I and CCL5-CCR1 dominated NK-related intercellular crosstalk, and unique transcription regulatory modules existed across NK subpopulations. Functional assays verified IGF1/IGF1R signaling dose-dependently promotes NK inflammatory cytokine release, cytotoxic gene expression, cell viability and target cell killing. These findings delineate dynamic NK subpopulation remodeling during SIRS and validate IGF1/IGF1R as a potential key pathway governing NK effector function, providing a transcriptomic basis for identifying candidate biomarkers and potential therapeutic targets in SIRS.

**Supplementary Information:**

The online version contains supplementary material available at https://doi.org/10.1007/s10238-026-02285-2.

## Introduction

Systemic inflammatory response syndrome (SIRS) is a clinical syndrome elicited by diverse etiological factors, including infection, trauma [1], craniocerebral injury [2], burns [3, 4] and pancreatitis. Its incidence exceeds 50% among patients in the intensive care unit (ICU), and it can provoke hypotension, organ failure and even death. During the development of SIRS, the inflammatory cascade induced by excessive release of cytokines and inflammatory mediators, together with activation of the complement system, ultimately leads to a series of pathological alterations such as leukocytosis and elevated microvascular permeability. In addition, the extensive release of inflammatory mediators, particularly cytokines, dampens the pain threshold in affected individuals. Suppression of the inflammatory response constitutes a crucial measure for the prevention of SIRS. Sepsis is a life‑threatening syndrome triggered by infection, frequently accompanied by SIRS, with high global mortality and morbidity [3, 5], yet no specific standardized therapeutic strategy is currently available for sepsis [6–8]. To date, the immune status of monocytes, lymphocytes and neutrophils in patients with SIRS and sepsis has been relatively well described [9–11]. However, as a pivotal cell type bridging innate and adaptive immunity, the immune characteristics and dynamic change patterns of natural killer (NK) cells in SIRS remain to be further elucidated.

Human peripheral blood NK cells display remarkable functional heterogeneity and can be mainly categorized into 2 major subpopulations: approximately 90% are CD56^dim^ CD16^bright^ NK cells characterized by enhanced cytotoxicity, while around 10% are CD56^bright^ CD16^−/dim^ NK cells with stronger proliferative capacity and higher production of cytokines including interferon-γ (IFN-γ) and tumor necrosis factor-α (TNF-α) [12–15]. Such functional heterogeneity endows NK cells with a distinct double-edged sword property. They can facilitate the resolution of inflammation by rapidly eliminating pathogens, but may also amplify systemic inflammatory responses under certain circumstances [16]. Therefore, in septic patients, circulating NK cells have been reported to be associated with both improved and worsened prognosis, and their exact role in acute inflammatory responses remains controversial without a unified conclusion.

Existing studies have preliminarily revealed the critical role of NK cells in sepsis. In experimental murine sepsis, NK cells exacerbate deleterious excessive inflammatory responses, which are mainly mediated by IL-15-induced IFN-γ production [17]; however, NK cell dysfunction also appears to contribute to the subsequent immunosuppressive state. Clinical studies further demonstrate that a sharp reduction in the number of peripheral blood NK cells occurs in septic patients within 24 h, whereas the underlying mechanisms remain incompletely elucidated and may be associated with increased apoptosis and migration toward infectious sites.

Collectively, accumulating evidence supports that NK cells exhibit stage-dependent and dynamically plastic functional characteristics throughout the course of sepsis. During the acute phase of sepsis, NK cells are hyperactivated and secrete abundant pro‑inflammatory cytokines including IFN‑γ and TNF‑α, which markedly aggravate systemic inflammation and even drive the formation of a cytokine storm, thereby causing tissue damage and organ dysfunction [18, 19]. Of note, NK cell‑derived granzymes mainly exert pro‑inflammatory roles in the early stage, rather than their canonical cytotoxic functions. As the disease progresses, the host immune status may shift from an early pro‑inflammatory peak to late‑phase immunosuppression. In this late phase, NK cells frequently undergo massive apoptosis and numerical depletion, accompanied by severely compromised function. Such NK cell deficiencies and functional anomalies are recognized as key hallmarks of systemic immunosuppression in late‑stage sepsis, resulting in impaired immune surveillance, elevated risks of secondary infection and latent viral reactivation [20], which further contribute to disease exacerbation, prolonged hospital stays and increased long‑term mortality.

Nevertheless, current studies still present obvious limitations. First, most evidence is obtained from highly heterogeneous patient cohort studies, and differences in individual underlying diseases may confound the genuine immune phenotype of NK cells, which urgently requires validation in standardized models. Second, previous investigations have not fully delineated the specific roles of distinct functional NK cell subpopulations (cytotoxic, immunotolerant, immunoregulatory, etc.) across different stages of SIRS, and precise technical approaches are urgently needed to dissect the dynamic changes and molecular regulatory networks of NK cells during SIRS. However, the development of single-cell RNA sequencing (scRNA-seq) has provided a novel perspective for systematically dissecting immune cell heterogeneity and functional status [21–24]. This technology allows the identification of immune cell subset composition, transcriptional characteristics, and key regulatory molecules at single-cell resolution [25–27]. Nonetheless, single-cell studies targeting NK cells in SIRS models remain incomplete. In particular, the dynamic transcriptomic alterations and molecular regulatory networks of NK cell subpopulations in the standardized inflammatory model induced by LPS have not been systematically reported.

In the present study, we analyzed public scRNA-seq datasets obtained from LPS‑induced SIRS in healthy volunteers. We systematically characterized the transcriptomic profiles of peripheral blood NK cells at the early (4 h) and late (Day 7) stages of SIRS, dissected dynamic alterations in subset proportions and functional status of NK cells across different stages, and identified key molecular markers governing the functional transition of NK cells. Our findings provide direct single-cell-level evidence for elucidating the mechanism underlying the transition from cytokine storm to immunosuppression.

## Materials and methods

### Data acquisition and processing

ScRNA-seq data were retrieved from the Gene Expression Omnibus (GEO; https://www.ncbi.nlm.nih.gov/geo/) under accession number GSE212092. The dataset employed for single-cell analysis comprised 9 peripheral blood mononuclear cell (PBMC) samples collected at 3 time points from 3 healthy donors with LPS-induced SIRS. The data were derived from publicly available databases, so ethical review was unnecessary for this study.

### Single-cell data processing

Raw gene expression data were imported into R (v4.4.1) for downstream analysis using the Seurat package (v4.3.0) [28, 29]. Quality control (QC) was performed to exclude low-quality cells and doublets: doublet detection was implemented with the DoubletFinder package (v2.0.3), and cells were retained based on the following QC thresholds: (1) number of detected genes (nFeature) between 300 and 6,000; (2) total unique molecular identifier (UMI) counts (nCount) between 500 and 100,000; (3) mitochondrial gene expression fraction (relative to total cellular UMI counts) ≤ 25%; and (4) erythroid gene expression fraction (relative to total cellular UMI counts) ≤ 5%.

Subsequently, the gene expression matrix of cells was generated by logarithmic normalization and linear regression techniques using the “NormalizeData” and “ScaleData” functions in the Seurat R package (v4.3.0). Firstly, the “NormalizeData” function was used for normalization processing [30]. Then, the “FindVariableFeatures” function was employed to select the top 2,000 highly variable genes (HVGs), and these genes were further standardized‌ using the “ScaleData” function. Batch effects were corrected using the Harmony package (v0.1.1) [31], and the first 30 principal components (PCs) were retained for subsequent downstream analyses.

### Cell type identification and Ro/e analysis

The first 30 PCs were selected for dimensionality reduction and clustering analysis. Following dimensionality reduction and clustering, the outputs were projected onto a two-dimensional (2D) space using the Uniform Manifold Approximation and Projection (UMAP) algorithm to facilitate cell type identification [32–34]. Cell type-specific markers were retrieved from the CellMarker database (http://xteam.xbio.top/CellMarker/). These markers were then used to annotate cell clusters, enabling the identification of distinct cell types and characterization of their distribution patterns and relative proportions. To further explore the inherent heterogeneity within NK cells, the NK cell population was re-clustered, and each resulting subcluster was annotated based on its distinct gene expression signature. Subsequently, we calculated Ro/e values to evaluate stage-specific enrichment of distinct cell populations, defined as the ratio of observed cell numbers to expected numbers (Ro/e =observed/expected) [35]. Chi-square tests were applied to compute predicted cell numbers for each cell type-stage group, with Ro/e > 1 indicating significant cell enrichment at the corresponding stage.

### Enrichment analysis of differential gene expression

Differentially expressed genes (DEGs) for each cell type were identified using the “FindAllMarkers” function (Seurat package), implemented via the Wilcoxon rank-sum test with default parameters. DEGs were filtered with strict thresholds: log2 fold change (LogFC) > 0.25 and expression in ≥ 25% of cells within the corresponding cluster. To elucidate the functional implications of these DEGs, functional enrichment analyses were performed using the clusterProfiler (v4.6.2) and SCP (v0.4.8) packages, leveraging gene sets from the Gene Ontology (GO) database [36] and the Molecular Signatures Database (MSigDB, accessed via Gene Set Enrichment Analysis [GSEA]) [37, 38]. And we also conducted Kyoto Encyclopedia of Genes and Genomes (KEGG) functional enrichment analysis with the aid of the clusterProfiler package (v4.6.2) [39].

### AUCell score

Signature activity scores were computed using the scgmt package (v0.0.3; https://github.com/ZhaoLabs-SJTU/scgmt) [40, 41]. For this analysis, the “AUCell” method was specified via the “method” parameter. AUCell is a well-established algorithm for quantifying gene set activity in scRNA-seq data: it takes a predefined gene set as input and generates a quantitative “activity score” reflecting the functional activity of the target gene set in each individual cell.

### Developmental trajectory inference

Cell differentiation trajectories were constructed using the Monocle package (v2.24.0) [42, 43]. Cell subpopulations were then ordered by pseudotime. Genes with synchronized expression changes along the pseudotime trajectory were identified and visualized as a pseudotime heatmap. The Slingshot package (v2.6.0) [44, 45] was employed to infer cell lineages and pseudotime. This tool uses a clustering-based minimum spanning tree to delineate lineage structures and fits branch curves to these lineages via simultaneous principal curves. The “getCurves” function was used to derive smoothed trajectory curves [46]. Cellular stemness was evaluated using the CytoTRACE (v0.3.3) [47, 48].

### Cell-Cell communication

Potential cell-cell interactions among different cell types were predicted using the CellChat package (v1.6.1) [49, 50], with a significance threshold set at a P-value cutoff of 0.05. Specifically, we utilized the “Identify Communication Patterns” function to assess the number of intercellular communication patterns, while the “netVisual_diffInteraction” function was employed to visualize the differences in communication strength between cells [51].

### Single-cell regulatory network inference and clustering (SCENIC) analysis

To identify the most prominently dysregulated transcription factors (TFs) across each cell subpopulation, single-cell regulatory network inference and clustering were performed using the SCENIC package (v1.3.1) in Python software [52–54]. Specifically, the GRNBoost algorithm was utilized to infer potential target genes for each TF. Subsequently, DNA motif analysis was conducted to identify direct binding targets of these TFs. Ultimately, regulon activity in individual cells was quantified via the AUCell method, and the top 5 TFs with the highest regulon activity scores were designated as key candidates.

### Cell culture

The human NK cell line NK-92 and the human chronic myelogenous leukemia cell line K562 were obtained from the American Type Culture Collection. NK-92 cells were cultured in Alpha Minimum Essential Medium (α-MEM; Gibco) supplemented with 12.5% fetal bovine serum (FBS; Gibco), 12.5% horse serum (HS; Gibco), 2 mM L-glutamine (Gibco), 0.2 mM inositol (Sigma-Aldrich), 0.1 mM 2-mercaptoethanol (Sigma-Aldrich), 0.02 mM folic acid (Sigma-Aldrich), 100 U/mL penicillin, and 100 µg/mL streptomycin (Gibco). Recombinant human interleukin-2 (rhIL-2; PeproTech) was added to the culture medium at a final concentration of 200 IU/mL to maintain NK-92 cell viability and functional activity. K562 cells were cultured in RPMI-1640 medium (Gibco) supplemented with 10% FBS and 1% penicillin-streptomycin solution. All cells were maintained at 37 °C in a humidified incubator containing 5% CO₂. Cell viability was routinely confirmed to exceed 90% using trypan blue exclusion before subsequent experiments.

### rhIGF1 treatment and experimental grouping

Recombinant human insulin-like growth factor-1 (rhIGF1) was purchased from PeproTech (Rocky Hill, NJ, USA). rhIGF1 was reconstituted in sterile phosphate-buffered saline (PBS) containing 0.1% bovine serum albumin (BSA; Sigma-Aldrich) according to the manufacturer’s instructions. Based on preliminary dose-screening experiments and previously reported concentrations used for immune-cell functional studies, rhIGF1 was administered at final concentrations of 25 ng/mL, 50 ng/mL, and 100 ng/mL, designated as rhIGF1-L, rhIGF1-M, and rhIGF1-H, respectively.

For the dose-response experiments, NK-92 cells were divided into 4 groups: Control, rhIGF1-L (25 ng/mL), rhIGF1-M (50 ng/mL), and rhIGF1-H (100 ng/mL). Cells were cultured in complete NK-92 medium supplemented with rhIL-2 and treated with rhIGF1 for the indicated time periods prior to downstream analyses. For ELISA, apoptosis assays, flow cytometry-based cytotoxicity assays, and qRT-PCR analyses, NK-92 cells were stimulated with rhIGF1 for 24 h. For CCK-8 assays, NK-92 cells were continuously cultured with rhIGF1 for up to 4 days, and cell viability was assessed at Day 0, Day 1, Day 2, Day 3, and Day 4.

For the mechanistic experiments, NK-92 cells were divided into 6 groups: Control, si-Control, si-IGF1R, rhIGF1, si-Control + rhIGF1, and si-IGF1R + rhIGF1. Based on the dose-screening results, 100 ng/mL rhIGF1 was selected for subsequent mechanistic studies. NK-92 cells were first transfected with siRNAs targeting IGF1R or negative control siRNA, followed by stimulation with rhIGF1 (100 ng/mL) before downstream analyses, including ELISA, qRT-PCR, apoptosis assays, flow cytometry-based cytotoxicity assays, and cell viability measurements.

### siRNA transfection

Small interfering RNAs targeting human IGF1R and the corresponding non-targeting control siRNA were synthesized by GenePharma (Shanghai, China). The detailed siRNA sequences are provided in Supplementary Table 1.

For siRNA-mediated knockdown, NK-92 cells were transfected with si-Control or si-IGF1R using Lipofectamine RNAiMAX Transfection Reagent (Invitrogen) according to the manufacturer’s instructions. After 6 h of transfection, cells were supplemented with fresh complete NK-92 culture medium containing recombinant human IL-2 and maintained under standard culture conditions. Cells were harvested 24–48 h after transfection for subsequent experiments. Knockdown efficiency was validated by quantitative real-time PCR (qRT-PCR) analysis of IGF1R expression. The siRNA exhibiting higher knockdown efficiency was selected for downstream functional experiments. For rhIGF1 supplementation experiments, transfected NK-92 cells were subsequently stimulated with rhIGF1 (100 ng/mL) prior to downstream analyses, including ELISA, qRT-PCR, apoptosis assays, cytotoxicity assays, and cell viability measurements.

### Cell viability assay

Cell viability was assessed using a Cell Counting Kit-8 assay (CCK-8; Dojindo Laboratories) according to the manufacturer’s instructions. For the rhIGF1 dose-response experiments, cells were treated with rhIGF1 at final concentrations of 25, 50, or 100 ng/mL. For IGF1R knockdown experiments, NK-92 cells were transfected with si-Control or si-IGF1R and then treated with rhIGF1 (100 ng/mL) as indicated. Cell viability was measured at Day 0, Day 1, Day 2, Day 3, and Day 4. The absorbance at 450 nm was measured using a microplate reader (BioTek Synergy H1, Agilent Technologies). Each experimental condition was performed in at least 5 technical replicates, and the experiment was independently repeated 3 times.

### ELISA assay

Culture supernatants were collected from NK-92 cells after the indicated treatments. The concentrations of human IFN-γ, TNF-α, IL-6, and IL-1β in the culture supernatants were measured using commercially available ELISA kits according to the manufacturers’ instructions. The following kits were used: Human IFN-γ Quantikine ELISA Kit (R&D Systems), Human TNF-α Quantikine ELISA Kit (R&D Systems), Human IL-6 Quantikine ELISA Kit (R&D Systems), and Human IL-1β/IL-1F2 Quantikine ELISA Kit (R&D Systems). The absorbance was measured at 450 nm using a microplate reader (BioTek Synergy H1, Agilent Technologies). To normalize cytokine secretion to cellular protein content, parallel cell pellets from the same treatment groups were lysed in RIPA buffer (Beyotime) supplemented with protease inhibitor cocktail (Roche). Total protein concentrations were determined using the Pierce BCA Protein Assay Kit (Thermo Fisher Scientific). Cytokine levels were normalized to the corresponding total protein content and expressed as pg/mg total protein. Each experiment was independently repeated at least 3 times.

### Quantitative real-time PCR analysis

Total RNA was extracted from NK-92 cells using TRIzol Reagent (Invitrogen) according to the manufacturer’s instructions. RNA concentration and purity were assessed using a NanoDrop 2000 spectrophotometer (Thermo Fisher Scientific). Complementary DNA (cDNA) was synthesized using the PrimeScript RT Reagent Kit with gDNA Eraser (Takara) according to the manufacturer’s protocol. Quantitative real-time PCR was performed using TB Green Premix Ex Taq II (Takara) on a QuantStudio 5 Real-Time PCR System (Applied Biosystems, Thermo Fisher Scientific). The mRNA levels of IGF1R, IFNG, TNF, IL1B, IL6, GZMB, PRF1, NKG7, and GNLY were examined. GAPDH was used as the internal reference gene. Relative gene expression was calculated using the 2^−ΔΔCt^ method. Primer sequences are listed in Supplementary Table 1. Each sample was analyzed in triplicate, and all experiments were independently repeated at least 3 times.

### NK-cell cytotoxicity assay

NK-cell cytotoxicity was first evaluated using a lactate dehydrogenase (LDH) release assay. K562 cells were used as target cells because of their high sensitivity to NK-cell-mediated lysis. NK-92 cells were used as effector cells and were pretreated with rhIGF1 at the indicated concentrations for 24 h. After treatment, NK-92 cells were harvested, washed, and co-cultured with K562 target cells in round-bottom 96-well plates (Corning Costar).

The LDH release assay was performed using the CyQUANT LDH Cytotoxicity Assay Kit (Invitrogen) according to the manufacturer’s instructions. Briefly, NK-92 effector cells and K562 target cells were co-cultured for 4 h at an effector-to-target (E: T) ratio of 10:1 unless otherwise indicated. Target-cell spontaneous LDH release, effector-cell spontaneous LDH release, target-cell maximum LDH release, and medium background controls were included for each experiment.

After co-culture, cell-free supernatant from each well was transferred to a new 96-well plate. LDH reaction mixture was added and incubated at room temperature protected from light according to the kit protocol. Absorbance was measured at 490 nm using a microplate reader (BioTek Synergy H1, Agilent Technologies). Specific cytotoxicity was calculated using the following formula: Specific cytotoxicity (%) = [(experimental LDH release − effector spontaneous release − target spontaneous release) / (target maximum release − target spontaneous release)] × 100. Each condition was performed in triplicate, and the experiment was independently repeated 3 times.

### Flow cytometry-based cytotoxicity assay

Flow cytometry-based cytotoxicity assays were performed to directly quantify dead K562 target cells after co-culture with NK-92 cells. K562 cells were labeled with CellTrace Violet Cell Proliferation Dye (Invitrogen) according to the manufacturer’s instructions to distinguish target cells from NK-92 effector cells. Briefly, K562 cells were washed with PBS and incubated with CellTrace Violet working solution. Labeled K562 target cells were seeded into round-bottom 96-well plates. NK-92 effector cells from the indicated treatment groups were added at an E: T ratio of 10:1 and co-cultured for 4 h at 37 °C in 5% CO₂. After co-culture, cells were collected, washed with staining buffer, and stained with 7-AAD Viability Staining Solution (BioLegend). Samples were analyzed using a BD FACSCanto II flow cytometer (BD Biosciences). Data were analyzed using FlowJo software (BD Biosciences). Each experiment was independently repeated 3 times.

### Annexin V/PI apoptosis assay

Cell apoptosis was assessed using an Annexin V-FITC/PI Apoptosis Detection Kit (BD Pharmingen) according to the manufacturer’s instructions. Samples were analyzed within 1 h using a BD FACSCanto II flow cytometer (BD Biosciences). Data were analyzed using FlowJo software (BD Biosciences). Early apoptotic cells were defined as Annexin V-positive and PI-negative cells, whereas late apoptotic cells were defined as Annexin V-positive and PI-positive cells. Total apoptotic cells were calculated as the sum of early and late apoptotic populations. Each experiment was independently repeated 3 times.

### Data Statistics

GSEA used the Kolmogorov-Smirnov (KS) test [55, 56]. For CellChat, the permutation test was employed to identify significant ligand-receptor (L-R) interactions. CytoTRACE utilized the Wilcoxon rank-sum test to compare scores across groups. In Monocle, generalized linear modeling (GLM) was applied to identify genes with temporal expression differences along the pseudotime trajectory. TFs were analyzed using context-dependent statistical tests to pinpoint cell population-specific regulons.

Statistical analyses for in‑vitro experiments were performed using GraphPad Prism software (v9.0). Data are presented as the mean ± standard deviation (SD) from at least 3 independent experiments unless otherwise indicated. For comparisons between 2 groups, an unpaired two-tailed Student’s t-test was used. For comparisons among multiple groups, one-way analysis of variance (ANOVA) followed by Tukey’s post hoc test was performed. For CCK-8 assays involving multiple groups and multiple time points, two-way ANOVA followed by Tukey’s multiple-comparisons test was used. A P value < 0.05 was considered statistically significant.

## Results

### Dynamic changes of different cell types during SIRS

**Fig. 1:**
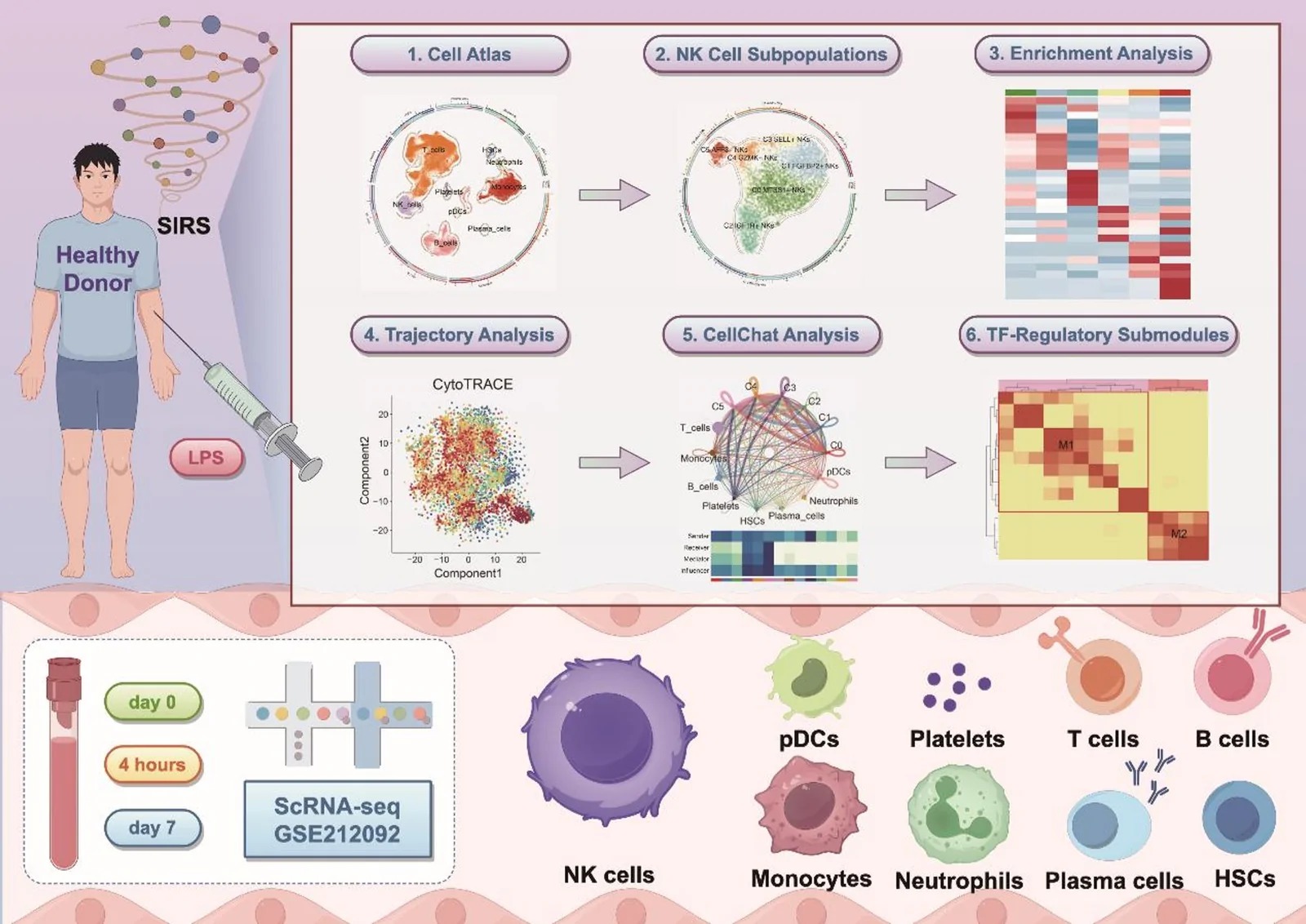
The analysis workflow of this study. We conducted single-cell sequencing analysis on the GSE212092 dataset to identify distinct NK cell subpopulations. Subsequently, through a series of bioinformatic analyses including enrichment analysis, pseudotime trajectory analysis, intercellular communication analysis, and TF regulatory analysis, we elucidated the functional significance of these NK cell subpopulations in SIRS

Figure 1 illustrated the overall workflow of this study. First, we obtained public datasets from the GEO database and conducted a series of analyses on single-cell data. To further elucidate the specific microscopic mechanisms of SIRS, we performed stringent QC and filtering on the acquired cells, retaining high-quality cells.

**Fig. 2:**
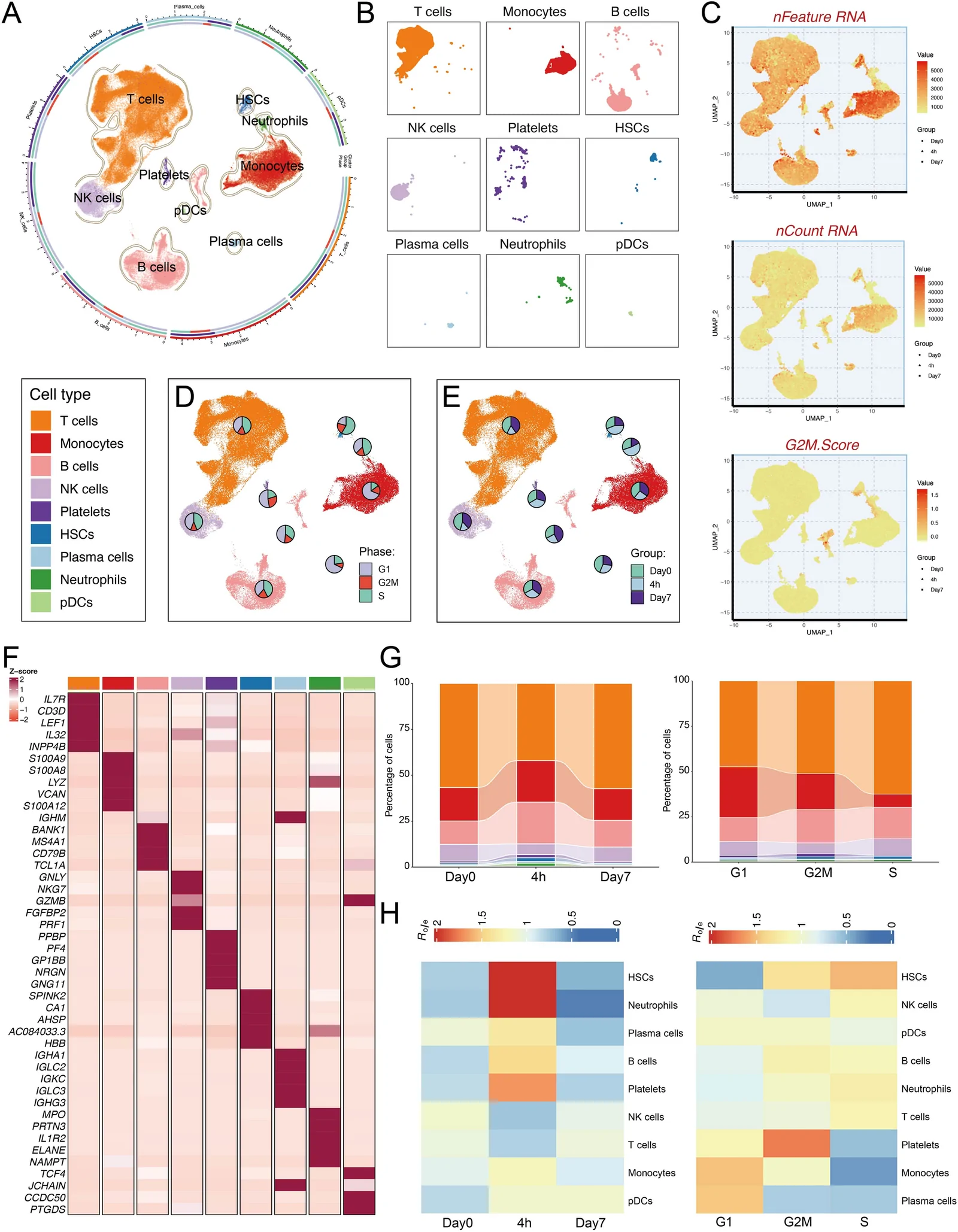
Single-cell characterization of 9 cell types. (**A**) The circular plot showed the clustering of 9 cell types, with individual cells colored according to their cell types. (**B**) The faceted UMAP plots illustrated the distribution characteristics of different cell types, respectively. (**C**) The UMAP plots displayed the values of nFeature RNA, nCount RNA and G2/M Score. (**D**) The UMAP plots demonstrated the distribution of different cell types. The pie charts overlaid on the UMAP plots showed the percentage of different cell types across 3 cell cycle phases (G1, G2/M, and S phases). (**E**) The pie charts overlaid on the UMAP plots exhibited the percentage of different cell types at 3 inflammation phases (Day 0, 4 h, and Day 7). (**F**) The heatmap presented the average expression levels of DEGs across different cell types. (**G**) Stacked bar plots showed the proportions of different cell types at different inflammation phases (Day 0, 4 h, and Day 7) and cell cycle phases (G1, G2/M, and S phases). (**H**) The Ro/e score was used to evaluate the preferences of each cell type at different inflammation phases (Day 0, 4 h, and Day 7) and different cell cycle phases (G1, G2M, and S phases)

Unsupervised clustering using the Seurat package identified 9 clusters, including T cells, monocytes, B cells, NK cells, platelets, hematopoietic stem cells (HSCs), plasma cells, neutrophils and plasmacytoid dendritic cells (pDCs) (Fig. 2a-b). Next, we used the heatmap to visualize the gene expression profiles of distinct cell clusters (Fig. 2f). We used UMAP plots to display the distribution characteristic value of nFeature RNA, nCount RNA and G2/M.Score across different cell types (Fig. 2c). The results showed that monocytes and HSCs had higher nFeature RNA and nCount RNA levels, while a few B cells had higher G2/M scores. These cells could be activated during the pathological progression of SIRS, potentially promoting disease development. Subsequently, we performed visualization analyses of the cell cycle and inflammation stages for these 9 cell types separately (Fig. 2d-e). We observed that monocytes and plasma cells were predominantly in the G1 phase. Neutrophils and HSCs were predominantly present in the early stage of inflammation, whereas T cells and NK cells were only detected in small quantities during this period. Next, we visualized the differences in the proportions of the 9 cell types across the 3 inflammation stages and 3 cell cycles (Fig. 2g), which led to a similar conclusion: the proportion of T cells and NK cells declined during the early stage of inflammation. Furthermore, the proportion of T cells was relatively high in the S phase. We further demonstrated the cell abundance of distinct cell clusters across different inflammation stages and cell cycles via Ro/e analysis (Fig. 2h). We found that the enrichment of NK cells varied significantly among different inflammation stages, with a lower enrichment observed in the early stage of SIRS. In contrast, HSCs and neutrophils were more enriched in the early stage of SIRS. Additionally, monocytes and plasma cells were enriched in the G1 phase of the cell cycle.

In conclusion, we analyzed the dynamic changes of different cells in SIRS and revealed the differences in gene expression profiles among distinct cell types. Collectively, these findings indicated that the dynamic alterations of immune cells may play a vital role in SIRS, and the proliferation and activity of NK cells may be suppressed in the early stage of SIRS.

### Functional characteristics and pathway regulation of cells in SIRS

**Fig. 3:**
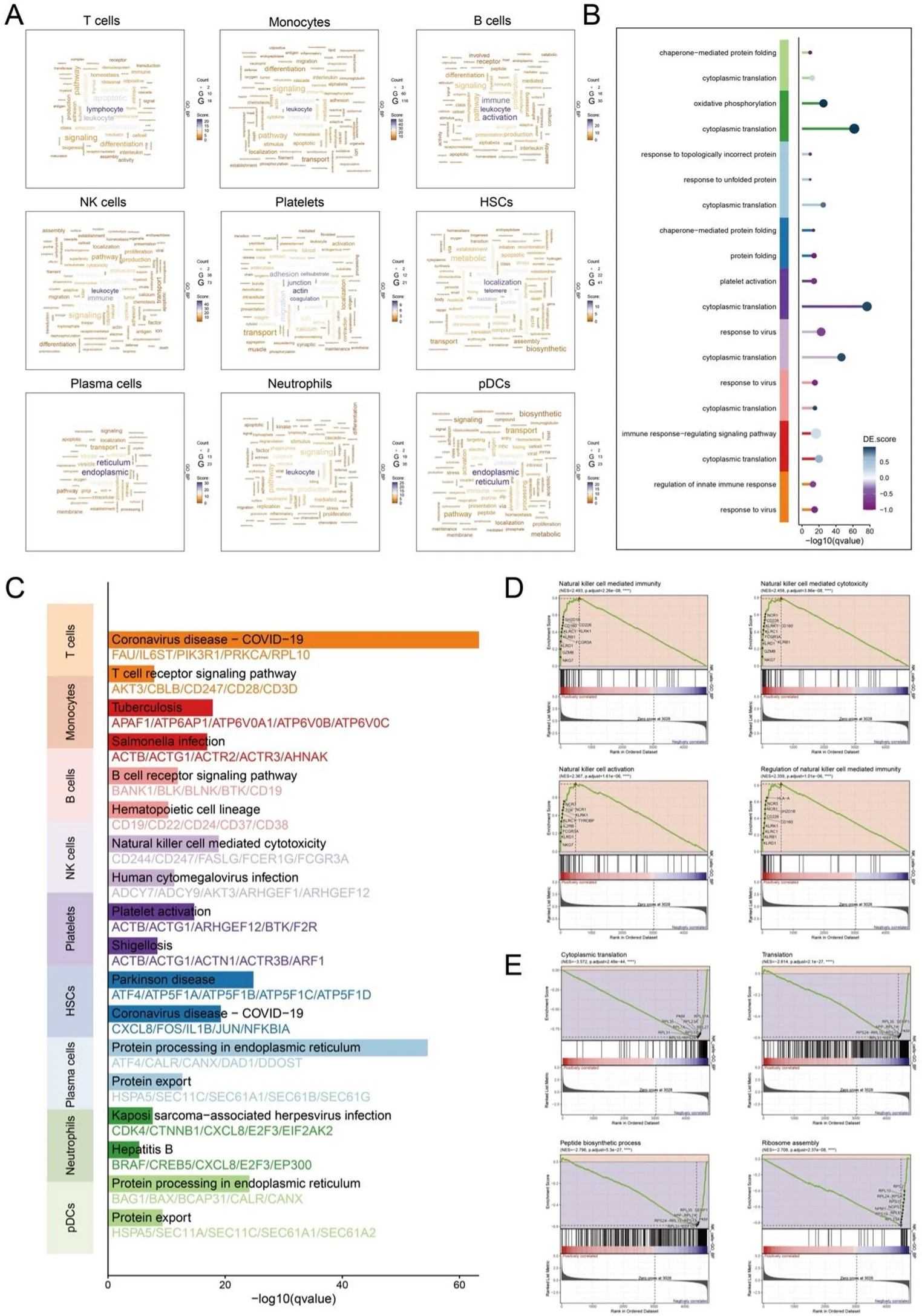
Functional enrichment profiles of DEGs in diverse cell types. (**A**) Word cloud plots displayed the outcomes of GOBP enrichment analysis according to the DEGs among different cell types. The font size indicated the quantity of genes, while the color indicated the enrichment score for each gene. (**B**) The GO enrichment analysis results for the DEGs among different cell types. (**C**) The KEGG enrichment analysis results for the DEGs among different cell types. (**D**-**E**) GSEA analyzed 8 positively and negatively enriched pathways in NK cells

Based on the DEGs, we conducted GO Biological Process (GOBP) enrichment analysis on highly expressed genes and visualized the results using word cloud diagrams based on the enrichment scores (Fig. 3a). T cells were associated with lymphocyte processes. Monocytes, NK cells and neutrophils were linked to leukocyte processes. B cells exhibited significant enrichment in activation, leukocyte and immune processes. Platelets were enriched in actin and junction processes. HSCs were enriched in localization and telomere processes. Plasma cells and pDCs were both enriched in endoplasmic and reticulum processes. The GO and KEGG enrichment analysis results for the DEGs across all cell types were summarized in Fig. 3b-c.

Further GSEA of NK cells was performed according to GOBP terms. The results showed that pathways such as NK cell mediated immunity, NK cell mediated cytotoxicity, NK cell activation, and regulation of NK cell mediated immunity showed a positive enrichment trend (Fig. 3d). In contrast, Fig. 3e showed that pathways such as cytoplasmic translation, translation, peptide biosynthetic process, and ribosome assembly showed a trend of negative enrichment in these NK cells, suggesting suppressed translational activity in SIRS-associated NK cells.

### Dynamic changes of different NK cell subpopulations during SIRS

**Fig. 4:**
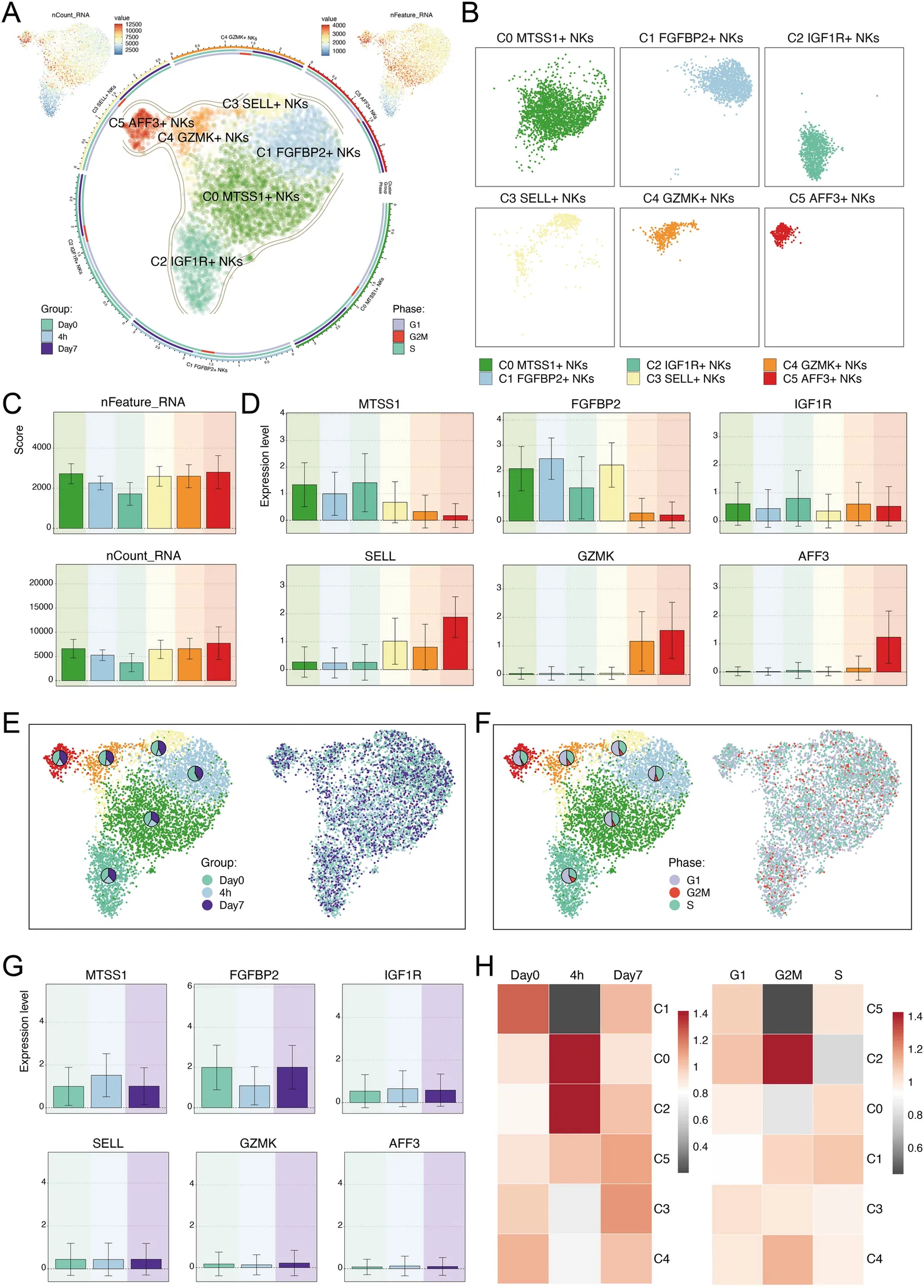
Single-cell characterization of NK cell subpopulations. (**A**) The circular plot showed the clustering of 6 NK cell subpopulations, with individual cells colored according to their cell types. The UMAP plots located at the top-left and top-right corners respectively displayed the nCount RNA and nFeature RNA values of different NK cell subpopulations. (**B**) The UMAP plots illustrated the distribution characteristics of the 6 NK cell subpopulations, respectively. (**C**) The bar plots compared the differences in nCount RNA and nFeature RNA scores among the 6 NK cell subpopulations. (**D**) The bar plots exhibited the expression profiles of 6 named genes across the 6 NK cell subpopulations. (**E**) The UMAP plots demonstrated the distribution patterns of different NK cell subpopulations and compared the distribution differences of NK cells derived from different groups. The pie charts showed the percentage of different NK cell subpopulations at 3 inflammation phases (Day 0, 4 h, and Day 7). (**F**) The pie charts overlaid on the UMAP plot displayed the percentage of the 6 NK cell subpopulations in 3 cell cycle phases (G1, G2M, and S phases). (**G**) The bar plot presented the expression profiles of 6 named genes at 3 inflammation phases (Day 0, 4 h, and Day 7). (**H**) The Ro/e score was applied to evaluate the preferences of the 6 NK cell subpopulations in different inflammation phases (Day 0, 4 h, and Day 7) and different cell cycle phases (G1, G2M, and S phases)

To elucidate the mechanism underlying the role of NK cells in SIRS, we performed further analyses. QC analysis revealed that the distributions of molecular counts (nCount_RNA) and detected gene numbers (nFeature_RNA) were uniform across all NK cell subpopulations (Fig. 4a, c). Based on the expression of MTSS1, FGFBP2, IGF1R, SELL, GZMK, and AFF3 (Fig. 4d), the NK cells were subdivided into 6 subpopulations, namely MTSS1⁺ NK cells (C0), FGFBP2⁺ NK cells (C1), IGF1R⁺ NK cells (C2), SELL⁺ NK cells (C3), GZMK⁺ NK cells (C4), and AFF3⁺ NK cells (C5) (Fig. 4a-b).

Subsequently, we analyzed the distribution of NK cell subpopulations across different disease stages. Compared with the Day 0 group (control), the enrichment of MTSS1⁺ NK cells (C0) and IGF1R⁺ NK cells (C2) were significantly elevated in the 4 h group (Fig. 4e, h). Both of these subpopulations exhibited lower enrichment in the control group (Day 0), highlighting their strong association with SIRS progression. In contrast, the C1 NK cell subpopulation underwent a sharp reduction at the 4 h time point. Cell cycle analysis further demonstrated that the C2 NK cell subpopulation was enriched in the G2/M phase (Fig. 4f, h), suggesting that it may be in an active proliferative state.

During the late immune suppression phase (Day 7), the enrichment of C0 and C2 NK cell subpopulations decreased significantly, while those of C3, C4, and C5 NK cell subpopulations relatively increased (Fig. 4e, h). Figure 4g showed the expression profiles of 6 named genes at 3 inflammation phases. Distinct inflammatory stages were characterized by unique immune cell composition profiles, indicating that LPS induces stage-specific immune responses.

### Functional characteristics and pathway regulation of NK cell subpopulations in SIRS

To gain insights into the biological functions of each NK cell subpopulation, we performed DEG analysis, GO enrichment analysis, and GSEA.

**Fig. 5:**
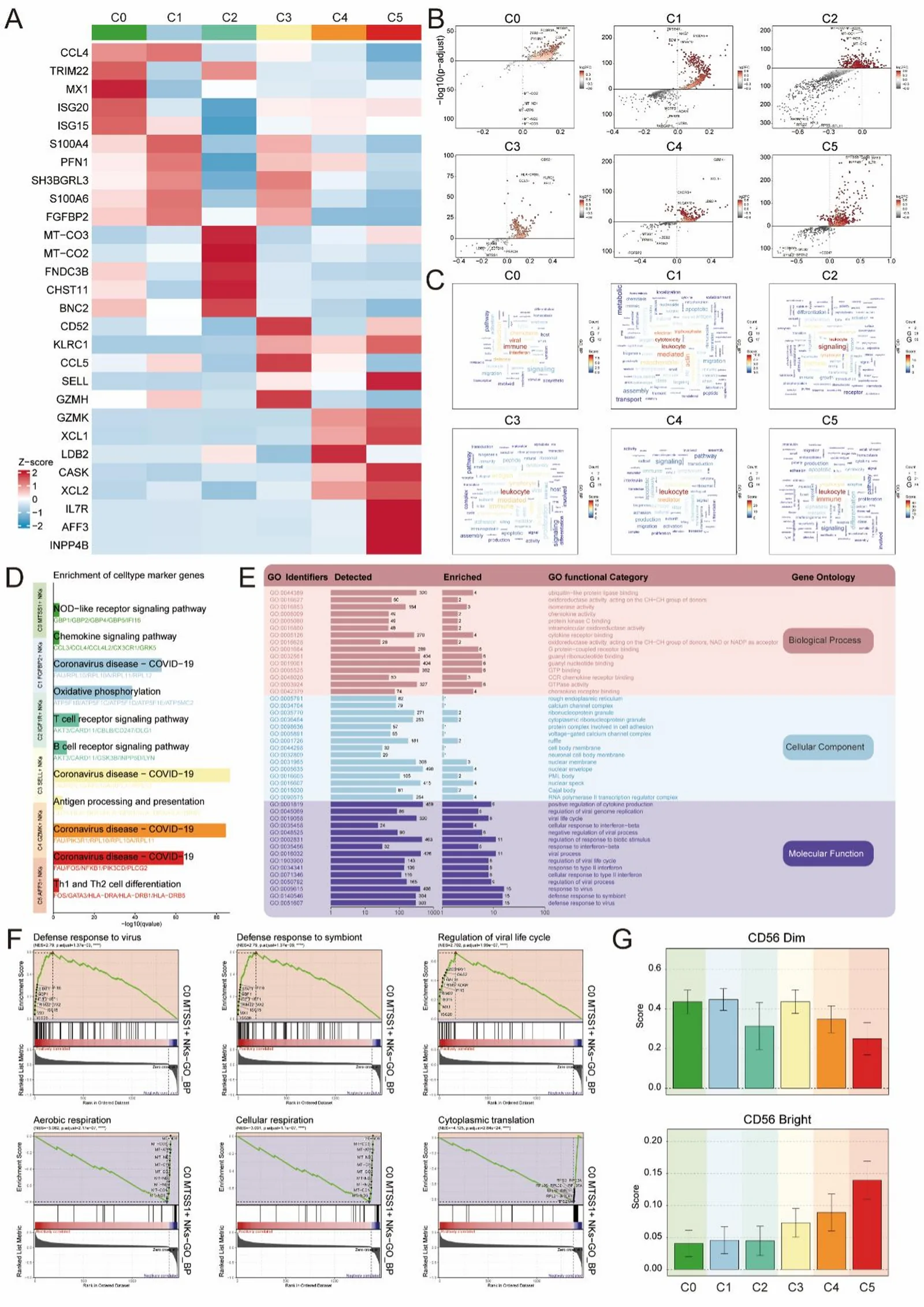
DEGs and functional enrichment analyses of NK cell subpopulations. (**A**) The heatmap showed the average expression levels of DEGs across the 6 NK cell subpopulations. (**B**) The volcano plot illustrated the top 5 significantly upregulated and top 5 downregulated genes among the 6 NK cell subpopulations. (**C**) The word cloud plot displayed the significantly enriched GO pathways in the 6 NK cell subpopulations. (**D**) The KEGG enrichment analysis demonstrated the significantly enriched pathways across the 6 NK cell subpopulations. (**E**) The GO enrichment analysis separately presented the significantly enriched status of the C0 NK cell subpopulation in the categories of GOBP, GO Cellular Component (GOCC), and GO Molecular Function (GOMF). (**F**) The GSEA analysis revealed the 6 pathways with positive and negative enrichment trends in the C0 NK cell subpopulation. (**G**) The bar plots presented the score of CD56^dim^ and CD56^bright^ across the 6 NK cell subpopulations

First, based on the heatmap of highly expressed DEGs across different NK cell subpopulations, the previously defined 6 NK cell subpopulations exhibited distinct transcriptomic profiles (Fig. 5a). Volcano plots further illustrated the DEGs of each NK cell subpopulation compared to all other NK cells, revealing the top 5 significantly upregulated and downregulated DEGs (Fig. 5b). In MTSS1⁺ NK cells (C0), interferon-stimulated genes (ISGs) such as MX1 and ISG15 were highly expressed, while mitochondrion-related gene MT-CO3 was downregulated, suggesting a Type I interferon (IFN-I) response.

Subsequently, we conducted GO enrichment analysis for each NK cell subpopulation and used word cloud plots to visually display the key pathway terms significantly enriched in different NK cell subpopulations (Fig. 5c). Furthermore, KEGG pathway enrichment analysis revealed that the marker genes of the C0 NK cell subpopulation were mainly enriched in the NOD-like receptor signaling pathway and chemokine signaling pathway (Fig. 5d). For the C0 NK cell subpopulation, its DEGs were predominantly enriched in NOD-like receptor signaling pathway and chemokine signaling pathway. For the C1 NK cell subpopulation, its DEGs were predominantly enriched in coronavirus disease-COVID-19 and oxidative phosphorylation. Meanwhile, the DEGs of the C2 NK cell subpopulation were mainly concentrated in the T cell receptor signaling pathway and B cell receptor signaling pathway. As for the C3 NK cell subpopulation, its DEGs were primarily enriched in coronavirus disease-COVID-19 and antigen processing and presentation. Notably, the DEGs of the C4 NK cell subpopulation were specifically enriched in coronavirus disease-COVID-19. Finally, the DEGs of the C5 NK cell subpopulation were mainly enriched in coronavirus disease-COVID-19 and Th1/Th2 cell differentiation. Given the core role of the C0 NK cell subpopulation in the acute inflammatory phase, we focused on its in-depth characterization. GO analysis indicated that the C0 NK cell subpopulation was specifically enriched in pathways such as positive regulation of cytokine production, cellular response to interferon-beta, and cellular response to type II interferon (Fig. 5e).

GSEA of MTSS1⁺ NK cells (C0) suggested that several pathways, including aerobic respiration, cellular respiration, and cytoplasmic translation, were suppressed, while defense response to virus, defense response to symbiont, and regulation of viral life cycle were activated (Fig. 5f).

Figure 5g demonstrated that the C0, C1, and C3 NK cell subpopulations exhibited the highest CD56^dim^ scores, whereas the C5 NK cell subpopulation showed the lowest scores. In contrast, the C5 and C4 NK cell subpopulations had the highest CD56^bright^ scores, while the C0-C2 NK cell subpopulations had the lowest. These data indicated that CD56^dim^ cells were associated with the C0, C1, and C3 NK cell subpopulations, whereas CD56^bright^ cells were correlated with the C4 and C5 NK cell subpopulations.

Collectively, these results suggest that the C0 NK cell subpopulation is a highly activated inflammatory effector subpopulation, with functions including rapid recognition of pathogenic stimuli, potent amplification of inflammatory signals, and potential regulation of adaptive immunity, thereby playing a crucial role in the early response to SIRS.

### Differentiation trajectories and heterogeneous characteristics of NK cell subpopulations

Differentiation constitutes a vital component of cellular function, particularly in the context of immune responses during SIRS. It endows cells with the capability to adapt and execute specialized functions, a property that may play a decisive role in determining disease outcomes.

**Fig. 6:**
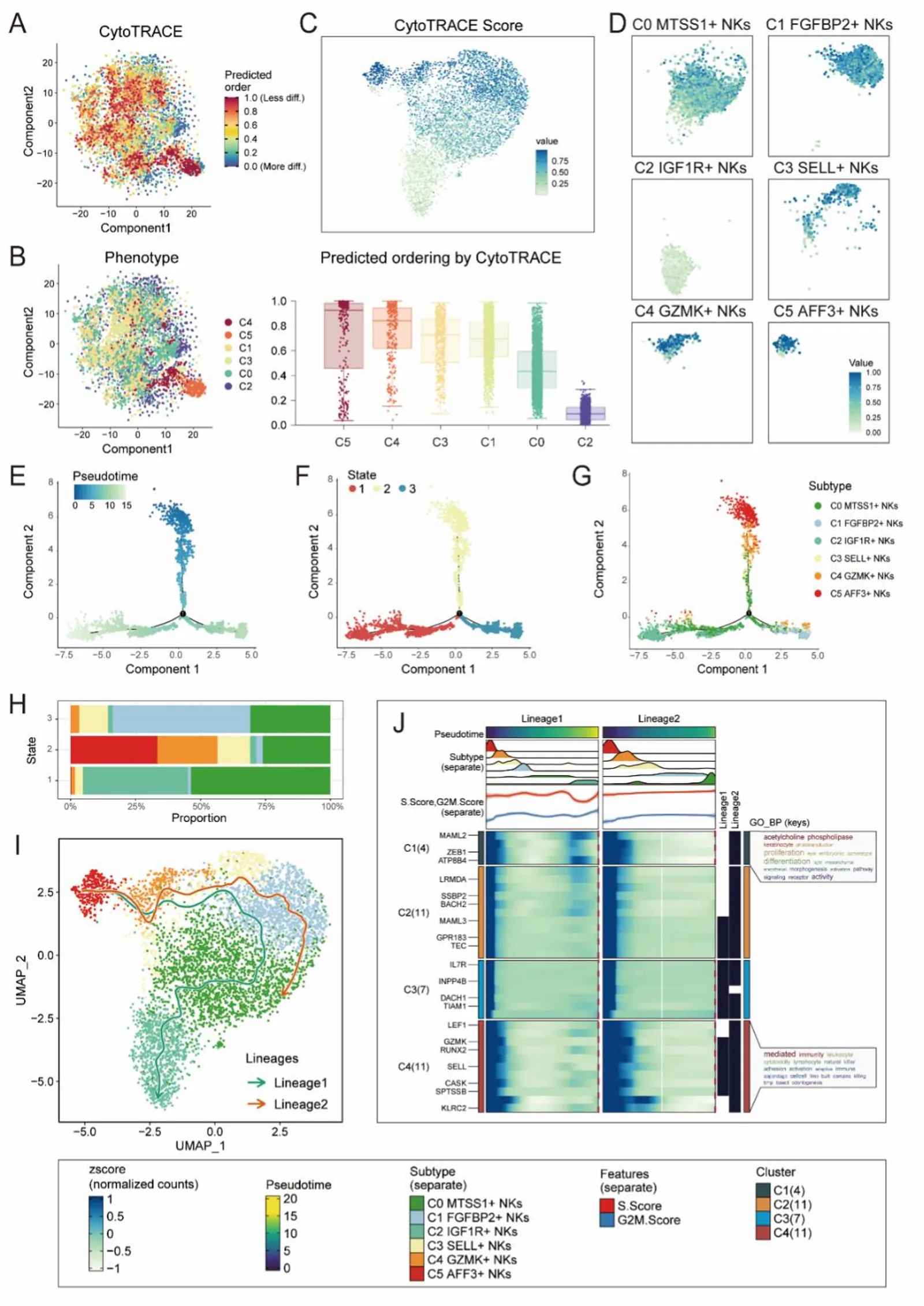
Differentiation features of NK cell subpopulations. (**A**) Visualization of CytoTRACE analysis results, showing the differentiation degree of different NK cell subpopulations: darker red indicates lower differentiation (higher stemness), while darker green indicates higher differentiation (lower stemness). (**B**) Left panel: Distribution of different NK cell subpopulations colored by subpopulation; Right panel: Boxplot showing CytoTRACE scores of each NK cell subpopulation, intuitively presenting the differentiation degree differences among subpopulations. (**C**) Visualization of CytoTRACE score distribution. The color gradient corresponds to the score level, showing the stemness-related score characteristics of the cell population. (**D**) CytoTRACE analysis facet map depicted the trajectory characteristics of each of the 6 NK cell subpopulations, with the color gradient corresponding to the relevant score value. (**E**) The pseudotime trajectory plot depicted the differentiation process of NK cell subpopulations. The color gradient corresponds to the progression of pseudotime. At branch point 1, the trajectory diverged into 2 distinct trajectories. (**F**) Cell state classification from trajectory analysis. 3 cell states (labeled with different colors) are identified, clearly showing the distribution of each state along the differentiation trajectory. (**G**) 2D pseudotime trajectory plot colored by the 6 NK cell subpopulations. (**H**) Stacked bar chart of NK subpopulation proportions across cell states: showing the proportion distribution of each NK cell subpopulation in State 1–3, reflecting the compositional differences of subpopulations in different differentiation states. (**I**) The UMAP plot displayed the slingshot differentiation trajectories of different NK cell subpopulations. (**J**) GOBP enrichment analysis of Lineage 1 and Lineage 2

We utilized CytoTRACE to assess the stemness levels of NK cell subpopulations. Via CytoTRACE visualization, the stemness levels of different NK cell subpopulations showed differences. C5 NK cell subpopulations had the highest stemness level (the lowest differentiation level), followed by C4 NK cell subpopulations and C3 NK cell subpopulations. Notably, C2 NK cell subpopulations displayed the lowest stemness scores (Fig. 6a-d). This finding is of particular significance, as it suggests that under physiological conditions, C5 NK cells may act as a reservoir of poorly differentiated cells, capable of generating more specialized cell types.

To further explore these distinct differentiative patterns, we employed Monocle to gain a more refined understanding of the differentiative trajectories. The starting points for this analysis were selected based on the lowest differentiation degrees identified by CytoTRACE. The results revealed that NK cells followed a simple trajectory featuring a single branching node and 3 states (Fig. 6f). The pseudotime trajectory originated from the upper region and bifurcated into 2 distinct branches: one extending to the left and the other to the right as it proceeded (Fig. 6e). Figure 6g illustrated the distribution of different NK cell subpopulations along the Monocle pseudotime trajectory, showing that the C1 and C2 NK cell subpopulations were less concentrated in the upper branch. The stacked proportion plot showed the compositional differences of subpopulations across the 3 cell states. The proportion of C2 NK cell subpopulations in State 1 was significantly higher, suggesting a closer association with this differentiation state (Fig. 6h). Furthermore, Slingshot analysis demonstrated that the C0 and C2 NK cell subpopulations were localized at the terminal ends of the differentiation branches (Fig. 6i), further validating the conclusions derived from the Monocle analysis. GOBP enrichment analysis of Lineage 1 and Lineage 2 indicated that the C5 NK cell subpopulation was enriched in cytotoxicity-related pathways, while the C2 NK cell subpopulation was enriched in pathways linked to the cell proliferation and differentiation (Fig. 6j). Collectively, these analytical investigations clarified the stemness properties, differentiation trajectories, as well as functional enrichment profiles of distinct NK cell subpopulations.

### Cell-cell communication in SIRS

To characterize the intercellular communication between NK cell subpopulations and other cell populations in SIRS, we employed CellChat to infer and dissect L-R interaction networks from single-cell transcriptomic data.

**Fig. 7:**
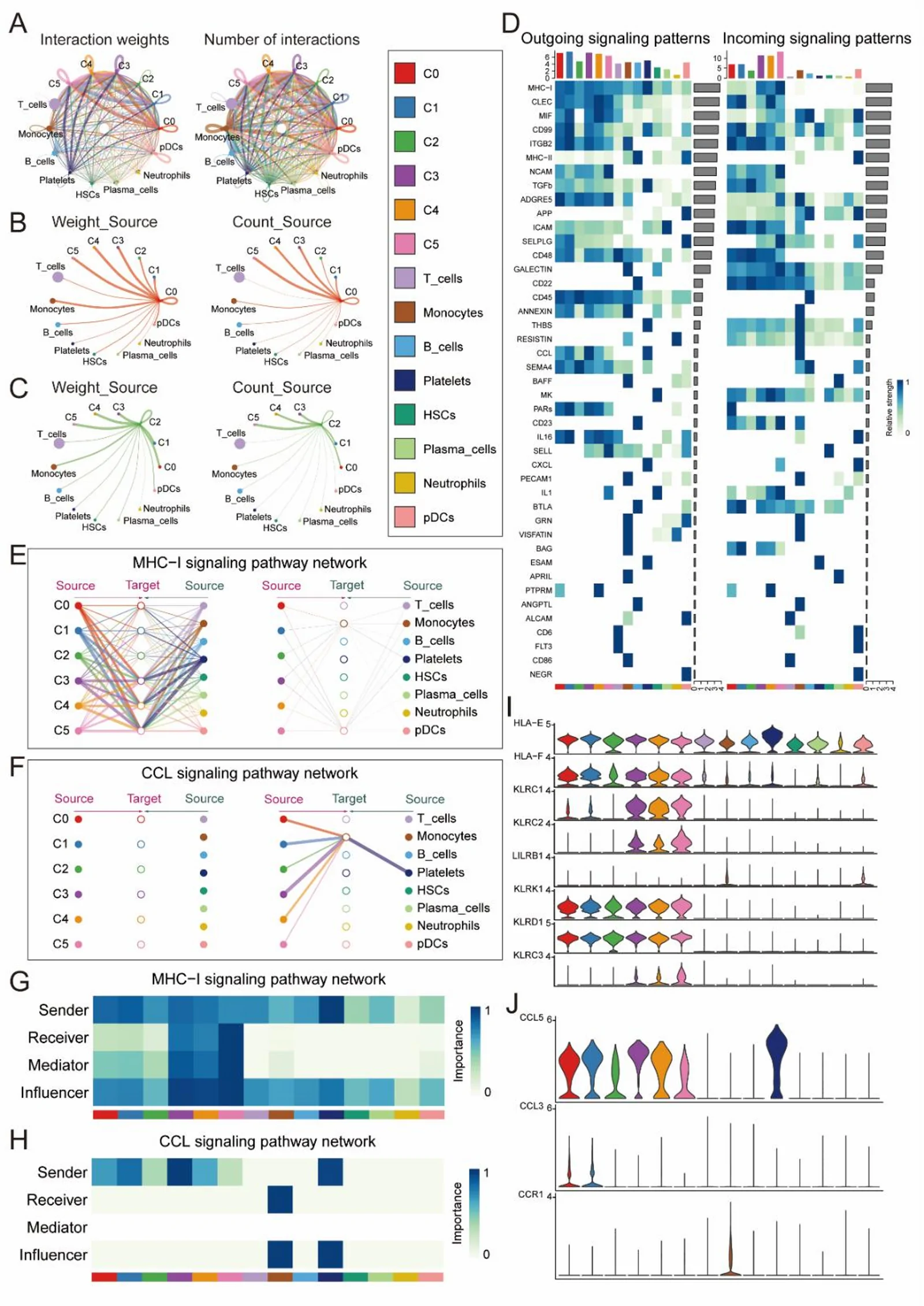
Intercellular communication profiles of NK cell subpopulations. (**A**) Intercellular communication network analysis plots for all cell types. The left plot displayed a network based on interaction weights (nodes represent cell types, including NK subpopulations C0-C5, T cells, monocytes, etc.), with edge thickness corresponding to interaction weights; the right plot displayed a network based on interaction counts, with edge thickness corresponding to the number of interaction events. The size of each circle was proportional to the number of cells in each group. (**B**) Circle plots displayed the intensity (left) and number (right) of interactions of C0 NK cell subpopulations as the source with other cells. (**C**) Circle plots displayed the intensity (left) and number (right) of interactions of C2 NK cell subpopulations as the source with other cells. (**D**) Heatmap of cellular signaling patterns. The left heatmap showed the outgoing signaling patterns of each cell type, where color depth was positively correlated with signal activity; The right heatmap showed the incoming signaling patterns of each cell type, presenting the signal-receiving characteristics of cells. (**E**-**F**) Hierarchical plot of MHC-I and CCL signaling pathways communication depicted the autocrine and paracrine interaction relationships between 6 NK cell subpopulations and other cells in the MHC-I signaling pathway, where filled circles represented source cells and open circles represented target cell types. (**G**-**H**) Centrality network regulation heatmap under the MHC-I and CCL signaling pathways illustrated the regulatory importance of different functional roles (e.g., signal senders, receivers) in this pathway, where color features corresponded to their centrality levels. (**I**-**J**) Violin plots showed proteins interacting between cells in the MHC-I and CCL signaling pathways

The intensity and number of interactions between all cell types from different samples were comprehensively summarized (Fig. 7a). The results showed that the interaction between 6 NK cell subpopulations and other cells (such as platelets and monocytes) was stronger. Next, the circle diagram was used to describe the intensity and quantity contribution of the interaction between cells by selecting the C0 NK cell subpopulation as the source (Fig. 7b), and the intensity and quantity contribution of the interaction between cells by selecting the C2 NK cell subpopulation as the source (Fig. 7c).

Then, in order to further analyze the key proteins governing intercellular incoming and outgoing signaling, we analyzed the contributions of distinct incoming communication patterns of target cells and outgoing communication patterns of secret cells, with results visualized in heatmap format (Fig. 7d). We found that in the incoming signaling patterns TGFb and ITGB2 signaling pathways contributed greatly to C2 NK cell subpopulations. In the outgoing signaling patterns, CCL and MHC-I signaling pathways contributed greatly to C3 NK cell subpopulations.

Given that all 6 NK subpopulations engaged in both incoming and outgoing communication via the MHC-I signaling network, we prioritized this pathway for in-depth investigation. Intriguingly, the C3, C4, and C5 NK cell subpopulations were capable of mediating both paracrine and autocrine interactions within the MHC-I pathway (Fig. 7e). The heatmap quantified the functional importance of each cell type (as senders, receivers, mediators, or influencers) within the MHC-I signaling network (Fig. 7g). The C3-C5 NK cell subpopulations exhibited markedly higher relative importance across all 4 functional roles in this pathway. The expression profiles of core signaling genes involved in the MHC-I pathway (including HLA-E, HLA-F, KLRC1, KLRC2, LILRB1, KLRK1, KLRD1, and KLRC3) were depicted in Fig. 7i. KLRC1, KLRC2, and KLRC3 were highly expressed in the C3, C4, and C5 NK cell subpopulations.

We then analyzed the CCL signaling pathway. Hierarchical plots revealed that monocytes were the sole target cell type in this pathway, while NK cells and platelets served as the primary signaling sources (Fig. 7f). Additionally, the C3 NK cell subpopulation primarily functioned as a signal sender in the CCL signaling pathway, while monocytes predominantly acted as signal receivers and influencers (Fig. 7h). The expression levels of CCL pathway-related signaling genes (CCL5, CCL3, and CCR1) are presented in Fig. 7j.

Collectively, our analyses unravel the complex intercellular interaction landscape between NK cell subpopulations and other cell populations in SIRS. MHC-I and CCL were important pathways for NK cells to interact with other cells, which were related to SIRS.

### Identification of TF-regulatory submodules in NK cell subpopulations

TFs exert direct genomic effects by binding to specific nucleotide sequences in the upstream regions of their target genes, modulating gene transcriptional activity and in turn sculpting the biological functions of cells [57]. Transcriptional regulatory modules are core molecular units that mediate cellular functional heterogeneity.

**Fig. 8:**
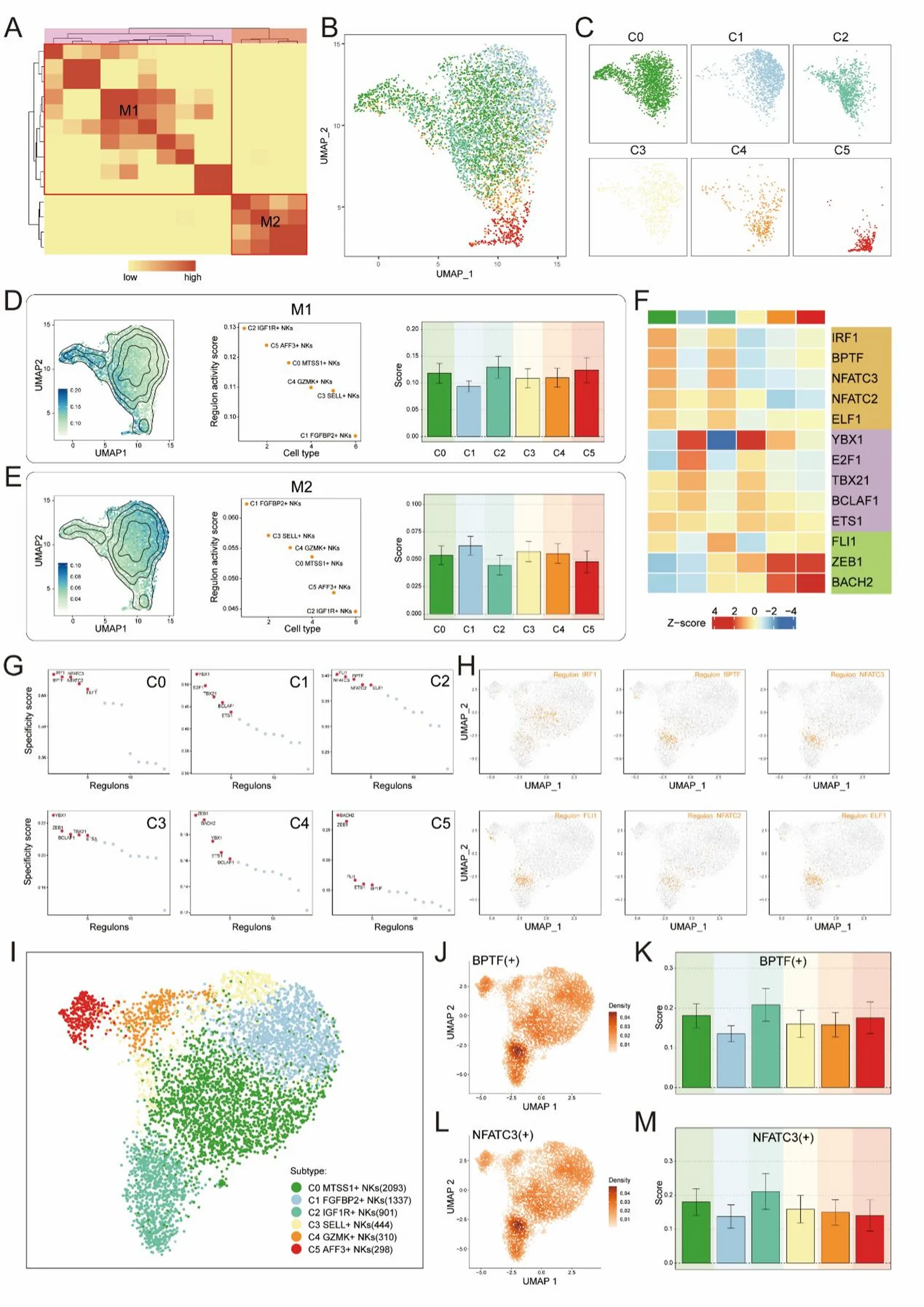
Regulatory modules and TF signatures of NK cell subpopulations. (**A**) Heatmap displayed the identification of 2 regulatory modules (M1 and M2) in NK cell subpopulations based on SCENIC regulatory rule modules and AUCell similarity scores. (B) UMAP plots colored and visualized all NK cells based on the activity scores of regulatory modules according to cell subpopulations. (**C**) Faceted UMAP plots of individual NK cell subpopulations based on the activity scores of regulatory modules. (**D**-**E**) Activity characteristic analysis of the M1 and M2 regulatory module. The left contour map showed the activity distribution of each module in the UMAP (color corresponds to activity level). The middle scatter plots displayed the regulatory activity of TFs in each module across different NK cell subpopulations. The right bar plot showed activity scores of each module across NK cell subpopulations. (**F**) The heatmap presented the top TFs for the 6 NK cell subpopulations. (**G**) Ranking of TFs in the NK cell subpopulations based on Regulon Specificity Score (RSS). (**H**) Distribution of top TFs in the C0 and C2 NK cell subpopulations based on RSS. (**I**) The UMAP plot showed that NK cells were divided into 6 different cell subpopulations. (**J**-**K**) Analysis of BPTF distribution and activity score. The UMAP plot visualized the distribution of BPTF. The bar plot depicted the AUC value of this TF across different NK cell subpopulations. (**L**-**M**) Analysis of NFATC3 distribution and activity score. The UMAP plot visualized the distribution of NFATC3. The bar plot depicted the AUC value of this TF across different NK cell subpopulations

First, we employed the connection specificity index (CSI) matrix to identify 2 distinct regulatory modules in NK cell subpopulations, designated as M1 and M2 (Fig. 8a). Next, we clustered the NK cells according to the activity of regulatory factors (Fig. 8b-c). We then focused on the activity profiling of the M1 and M2 regulatory modules. We visualized the activity of these 2 modules using UMAP plots, scatter plots, and bar graphs (Fig. 8d-e), and systematically analyzed the differences in regulatory activity of each module across different NK cell subpopulations. In the M1 regulatory module, C2, C5 and C0 NK cell subpopulations were more active. In contrast, C1, C3 and C4 NK cell subpopulations were more active in the M2 regulation module, suggesting that M1 and M2 modules may respectively regulate the functional phenotypes of different NK subpopulations. To clarify the core regulatory TFs governing each NK cell subpopulation, we identified the key TFs for each subpopulation and generated a heatmap to display the expression profiles of these top TFs across the 6 NK cell subpopulations (Fig. 8f). Key regulons with elevated specificity scores were highlighted, representing the distinct regulatory features of the C0-C5 NK cell subpopulations (Fig. 8g). Notably, some TFs were among the core regulatory factors in multiple subpopulations, such as YBX1, which was one of the top 5 key TFs in C1, C3, and C4 NK cell subpopulations, suggesting that it may be involved in regulating the common biological processes of NK cell subpopulations; while other TFs showed subpopulation preference, such as IRF1, which was only a core regulatory factor in the C0 NK cell subpopulation, implying that it may mediate the specific functions of the C0 NK cell subpopulation. The distribution of representative TFs in C0 and C2 NK cell subpopulations was illustrated in the UMAP plots (Fig. 8h). Further analysis of the core TFs BPTF and NFATC3 revealed that BPTF(+) and NFATC3(+) were more prominently distributed in the C2 NK cell subpopulation and C0 NK cell subpopulation (Fig. 8i-m), suggesting these TFs were specific regulators of the corresponding subpopulations.

### RhIGF1 enhances inflammatory activation and cytotoxic programs in NK-92 cells

To investigate whether exogenous IGF1 regulates NK-cell activation and effector function, NK-92 cells were treated with increasing concentrations of recombinant human IGF1 (rhIGF1; 25, 50, and 100 ng/mL). Since activated NK cells are characterized by enhanced inflammatory cytokine secretion, increased cytotoxicity, and elevated expression of cytotoxic effector molecules, we subsequently evaluated cytokine production, target-cell killing capacity, cell viability, and transcriptional alterations associated with NK-cell activation.

**Fig. 9:**
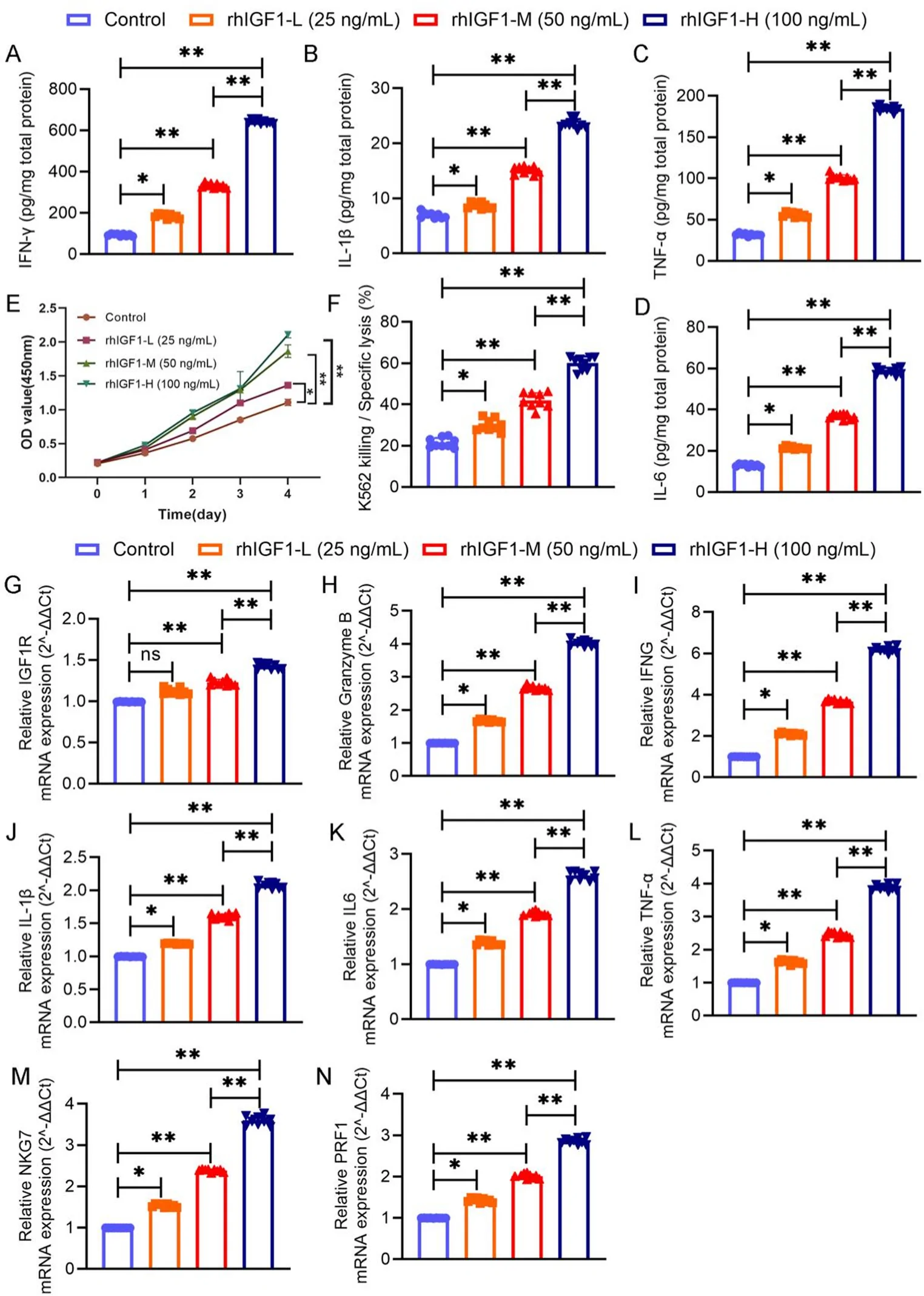
rhIGF1 promotes inflammatory activation and cytotoxic phenotypes in NK-92 cells. (**A**-**D**) ELISA analysis of IFN-γ, IL-1β, TNF-α, and IL-6 levels in culture supernatants. Cytokine concentrations were normalized to total protein content. (**E**) CCK-8 assays showing NK-92 cell viability from Day 0 to Day 4 after rhIGF1 treatment. (**F**) LDH-based cytotoxicity assays showing NK-92-mediated killing of K562 target cells. (**G**-**N**) qRT-PCR analysis of IGF1R, GZMB, IFNG, IL1B, IL6, TNF, NKG7, and PRF1 mRNA levels in NK-92 cells after rhIGF1 treatment. Gene expression levels were normalized to GAPDH and calculated using the 2^−ΔΔCt^ method. Data are presented as mean ± SD from at least 3 independent experiments. *P < 0.05, **P < 0.01

We first examined inflammatory cytokine secretion by ELISA. RhIGF1 treatment significantly increased IFN-γ and TNF-α secretion in a dose-dependent manner compared with the control group (Fig. 9a-d). IL-1β and IL-6 levels were also moderately elevated following rhIGF1 stimulation. Among these cytokines, IFN-γ exhibited the most prominent increase, indicating enhanced inflammatory activation of NK-92 cells after IGF1 stimulation.

Because inflammatory activation of NK cells is frequently accompanied by enhanced cytotoxic function, we next assessed NK-cell-mediated target-cell killing using an LDH-based cytotoxicity assay. K562 cells were selected as target cells because they are highly sensitive to NK-cell-mediated lysis and are widely used for evaluating NK-cell cytotoxic activity. rhIGF1 treatment significantly enhanced NK-92-mediated killing of K562 cells in a concentration-dependent manner (Fig. 9f), suggesting that IGF1 promotes NK-cell cytotoxic function.

To determine whether the enhanced effector phenotype was associated with altered cellular viability, CCK-8 assays were performed over a 4-day period. Since NK-cell activation and sustained cytotoxicity require adequate cellular survival and metabolic activity, NK-92 cell viability was evaluated following rhIGF1 stimulation. rhIGF1 treatment significantly increased NK-92 cell viability over time, with the strongest effect observed in the rhIGF1-H group at Day 4 (Fig. 9e). These findings indicate that IGF1 enhances NK-cell viability in a dose- and time-dependent manner.

To further characterize the molecular alterations induced by rhIGF1, qRT-PCR analyses were performed to evaluate inflammatory and cytotoxic effector genes. IFNG and TNF were selected as transcriptional markers of NK-cell inflammatory activation, whereas GZMB, PRF1, and NKG7 were analyzed because they are critical mediators of NK-cell cytotoxic granule release and target-cell killing. rhIGF1 treatment significantly increased the expression of IFNG, TNF, IL1B, and IL6 compared with the control group (Fig. 9i-l). Moreover, GZMB, PRF1, and NKG7 were markedly upregulated following rhIGF1 stimulation (Fig. 9h, m-n), indicating enhanced cytotoxic transcriptional programs in NK-92 cells. IGF1R expression showed only a modest increase after rhIGF1 treatment (Fig. 9g), suggesting that the observed biological effects were primarily associated with downstream activation of IGF1R signaling rather than major transcriptional induction of the receptor itself.

Collectively, these findings demonstrate that rhIGF1 enhances inflammatory activation, cellular viability, and cytotoxic effector programs in NK-92 cells in a dose-dependent manner.

### IGF1R knockdown attenuates rhIGF1-induced inflammatory activation and cytotoxic phenotypes in NK-92 cells

**Fig. 10:**
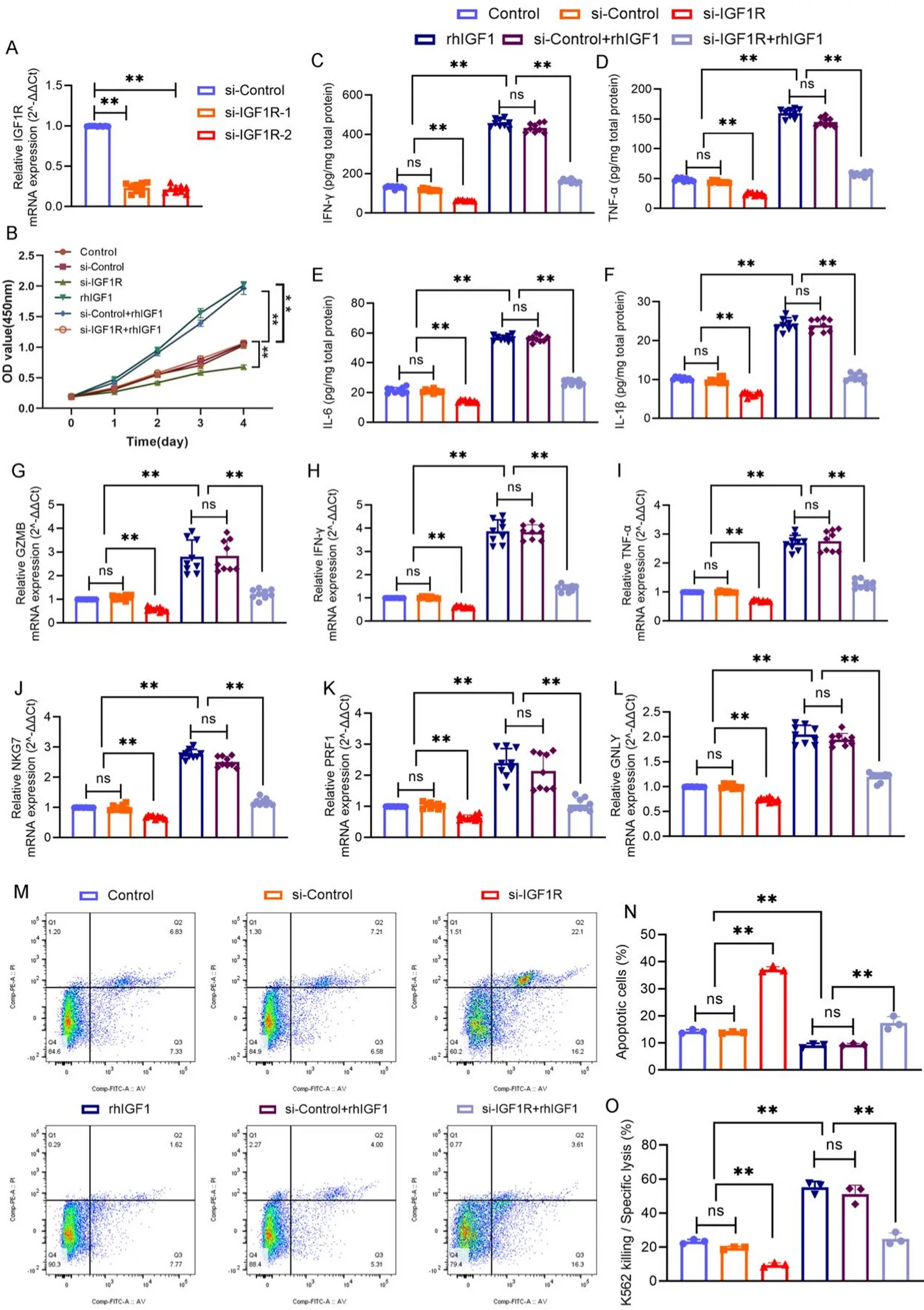
IGF1R knockdown attenuates rhIGF1-induced inflammatory activation and cytotoxic function in NK-92 cells. (**A**) qRT-PCR analysis of IGF1R knockdown efficiency in NK-92 cells transfected with 2 independent siRNAs targeting IGF1R (si-IGF1R-1 and si-IGF1R-2). (**B**) CCK-8 assays showing NK-92 cell viability from Day 0 to Day 4 following IGF1R knockdown and rhIGF1 treatment. (**C**-**F**) ELISA analysis of IFN-γ, TNF-α, IL-6, and IL-1β secretion in culture supernatants. Cytokine concentrations were normalized to total protein content. (**G**-**L**) qRT-PCR analysis of GZMB, IFNG, TNF, NKG7, PRF1, and GNLY mRNA levels in NK-92 cells after IGF1R silencing and rhIGF1 stimulation. Gene expression levels were normalized to GAPDH and calculated using the 2^−ΔΔCt^ method. (**M**) Representative Annexin V-FITC/PI flow cytometry plots showing apoptosis in NK-92 cells under the indicated conditions. (**N**) Quantification of apoptotic NK-92 cells. Total apoptotic cells were calculated as the sum of early apoptotic and late apoptotic populations. (**O**) Flow cytometry-based cytotoxicity assay showing the percentage of dead K562 target cells after co-culture with NK-92 cells under the indicated conditions. Data are presented as mean ± SD from at least 3 independent experiments. ns, not significant; **P < 0.01

To further determine whether the pro-inflammatory and cytotoxic effects induced by rhIGF1 were dependent on IGF1R signaling, IGF1R was silenced in NK-92 cells using 2 independent siRNAs. qRT-PCR analysis demonstrated that both si-IGF1R-1 and si-IGF1R-2 efficiently reduced IGF1R expression compared with the si-control group, confirming successful knockdown efficiency (Fig. 10a). si-IGF1R-2, which exhibited slightly higher silencing efficiency, was selected for subsequent functional experiments.

To evaluate the effect of IGF1R knockdown on NK-cell viability, CCK-8 assays were performed over a 4-day period. Silencing of IGF1R significantly reduced NK-92 cell viability compared with the control and si-control groups, whereas rhIGF1 treatment markedly enhanced cell viability (Fig. 10b). Importantly, IGF1R knockdown substantially attenuated the rhIGF1-induced increase in viability, indicating that the pro-survival effect of rhIGF1 is largely dependent on IGF1R signaling.

Because NK-cell activation is characterized by enhanced inflammatory cytokine production, ELISA assays were subsequently performed to quantify cytokine secretion. rhIGF1 treatment significantly increased IFN-γ, TNF-α, IL-6, and IL-1β secretion compared with the control group (Fig. 10c-f). In contrast, IGF1R silencing markedly suppressed cytokine production. Moreover, the elevated cytokine secretion induced by rhIGF1 was partially reversed following IGF1R knockdown. Among these cytokines, IFN-γ and TNF-α exhibited the most pronounced alterations, further supporting a critical role for the IGF1/IGF1R axis in regulating NK-cell inflammatory activation.

To further characterize the molecular alterations associated with IGF1R signaling, qRT-PCR analyses were performed to assess inflammatory and cytotoxic effector genes. Consistent with the cytokine secretion results, rhIGF1 stimulation significantly increased the expression of IFNG and TNF, whereas IGF1R silencing markedly reduced their expression levels (Fig. 10h-i). Furthermore, the cytotoxicity-associated genes GZMB, NKG7, PRF1, and GNLY were significantly upregulated following rhIGF1 treatment but were substantially suppressed after IGF1R knockdown (Fig. 10g, j-l). Importantly, IGF1R silencing markedly attenuated the rhIGF1-induced upregulation of these cytotoxic genes, suggesting that IGF1-mediated enhancement of NK-cell effector programs is dependent on IGF1R signaling.

Because IGF1R signaling is closely associated with cellular survival pathways, Annexin V/PI flow cytometric analysis was further performed to assess apoptosis. IGF1R knockdown significantly increased the proportion of apoptotic NK-92 cells compared with the control and si-control groups (Fig. 10m-n). In contrast, rhIGF1 treatment reduced apoptotic cell death, whereas the anti-apoptotic effect of rhIGF1 was partially reversed following IGF1R silencing. These findings indicate that IGF1R signaling contributes to the maintenance of NK-cell survival.

To directly assess NK-cell cytotoxic function, K562 target cells were co-cultured with NK-92 cells under the indicated conditions, followed by flow cytometric quantification of dead target cells. rhIGF1 treatment significantly enhanced NK-92-mediated killing of K562 cells, whereas IGF1R knockdown markedly impaired this cytotoxic effect. Moreover, IGF1R silencing substantially attenuated rhIGF1-induced enhancement of K562 killing (Fig. 10o), indicating that IGF1R is required for IGF1-mediated augmentation of NK-cell cytotoxic activity.

Collectively, these findings demonstrate that IGF1R is required for rhIGF1-induced inflammatory activation, survival, and cytotoxic function in NK-92 cells, supporting a functional role for the IGF1/IGF1R axis in regulating NK-cell effector phenotypes.

## Discussion

This study aimed to further characterize the immune status of NK cells in SIRS. Previous flow cytometry‑based studies demonstrated that the absolute counts of both CD56^bright^ and CD56^dim^ NK cell subpopulations in peripheral blood were significantly lower in patients with sepsis and SIRS within 24 h of suspected infection than in healthy individuals, regardless of the etiology of SIRS (infectious vs. non-infectious). Meanwhile, the proportion of CD56^dim^ NK cells in total lymphocytes was also markedly reduced in both patient groups, which is consistent with the lymphopenia commonly observed in ICU patients [58]. In line with previous reports [58], we observed a significant decrease in the proportion of circulating NK cells during SIRS, particularly in the C1 (FGFBP2+) NK cell subpopulation. This reduction represents a manifestation of systemic lymphopenia, which may result from the migration of NK cells to infectious sites [59] or the induction of apoptosis [60]. Notably, several studies have indicated that the proportion or count of NK cells is associated with patient prognosis [61]. The present study further confirmed that the distribution of NK cell subpopulations is altered during SIRS, with the C0 and C2 NK cell subpopulations becoming the relatively predominant NK cell populations in the early stage of SIRS.

Differential gene expression analysis revealed that the C0 NK cell subpopulation highly expressed FCGR3A (CD16), MX1, and ISG15, and exhibited the lowest CD56^bright^ score, suggesting that cytotoxic NK cells may shift toward a dysfunctional and hyperactivated CD56^−^ CD16^+^ phenotype in the early stage of SIRS—a transient phenotype previously described in HIV‑infected patients [62]. Studies have demonstrated the existence of a minor CD56^−^ NK cell subpopulation in peripheral blood, which expands during certain chronic viral infections. The C2 NK cell subpopulation, which expanded in the early stage of SIRS, displayed low scores for both CD56^bright^ and CD56^dim^, and may be more similar to the CD56^−^ NK cell subpopulation. The proportion of this NK cell subpopulation gradually decreases over time. The C1 NK cell subpopulation was the most abundant NK cell population in peripheral blood at baseline, likely corresponding to the NK1 cell subpopulation reported in previous studies [63]. It exhibited the highest CD56^dim^ score and highly expressed cytotoxic effector molecules (GZMA, GZMB) as well as CCL4.

The C5 NK cell subpopulation exhibited the highest CD56^bright^ score and harbored characteristic transcriptional signatures, including high expression of cytokine receptors (IL7R, IL18R1), granzyme K (GZMK), a distinct chemokine profile (XCL1, XCL2), cell surface markers (CD44, SELL, KLRC1), and the TF TCF1 (encoded by TCF7). This subpopulation corresponds to the NK2 cell subpopulation reported in previous studies [63] or the resting subpopulation that maintains basal defense, which features high expression of KLRC1 and NCAM1 [64]. The C3 NK cell subpopulation was more closely related to the NK3 cell subpopulation or adaptive subpopulation described in other studies, characterized by upregulation of CD2, KLRC2 (NKG2C), CCL5, GZMH, and CD3E, together with a prominent cytotoxicity score. NKG2C is encoded by the KLRC2 gene. NKG2C forms an obligate heterodimer with CD94 and binds to HLA-E, a non-classical MHC-I molecule. NKG2C+ adaptive NK cells undergo clonal expansion in response to viral infections, including cytomegalovirus and hantavirus. Consistent with previous studies, the C4 NK cell subpopulation displayed upregulation of KLRC1 (NKG2A, an inhibitory receptor) and exhibited CD56 expression intermediate between that of NK2 (C5 NK cell subpopulation) and NK1 (C1 NK cell subpopulation), potentially acting as a bridge between early and mature effector subpopulations. Nevertheless, the biological functions of each subpopulation in the NK cells remain to be further elucidated by experimental validation.

NK cell differentiation represents a gradual transition from the CD56^bright^ subpopulation to the CD56^dim^ subpopulation [65]. Our stemness score analysis demonstrated that the C5 NK cell subpopulation, which displayed the highest CD56^bright^ score, also exhibited the highest stemness score (indicating the lowest differentiation level). Modulating the differentiation status of NK cells may reshape their cytotoxicity and cytokine-secreting potential, thereby improving the prognosis of SIRS. Therefore, further in-depth exploration of the differentiation mechanisms of NK cells will advance our understanding of their functional roles in SIRS and provide theoretical support for the development of novel immunotherapeutic strategies.

The C3 and C5 NK cell subpopulations became the predominant populations on day 7 of SIRS. Cell communication analysis revealed that the MHC‑I signaling pathway plays a critical role in cellular interactions during SIRS, with C3, C4, and C5 as the major influencers of this pathway. Since activated T cells usually express higher levels of activating ligands (NKG2D) and lower levels of inhibitory ligands (MHC‑I) [66], NK cells can recognize and eliminate activated T cells through cytotoxicity, thereby modulating adaptive immune responses. Thus, C3, C4, and C5 NK cell subpopulations at the late stage of SIRS may serve important functions in restricting excessive immune reactions. In addition, the CCL signaling pathway, particularly the CCL5-CCR1 axis, dominates the crosstalk between the C3 NK cell subpopulation (acting as the sender) and monocytes (acting as the recipient). Following the onset of SIRS, the C3 NK cell subpopulation may secrete CCL5 and consequently recruit CCR1^+^ monocytes.

During the transition of NK cells between these functionally distinct developmental stages, extensive transcriptional and epigenetic reprogramming takes place. Investigating the functions of TFs in human NK cells will facilitate the identification of gene regulatory networks critical for the clinical enhancement of NK cell activity. Numerous positive regulators are novel and lack well‑established roles in human NK cell function. At present, our understanding of the specific TFs governing the function of mature human NK cells remains limited. Further experiments are warranted to determine whether these TFs act as potential drivers of chromatin accessibility in NK cell subpopulations.

A particularly noteworthy finding of our study is the identification of the C2 IGF1R + NK cell subpopulation, which was defined by high expression of IGF1R and emerged as one of the 2 dominant subpopulations during the early inflammatory phase (4 h) of SIRS. Compared with baseline (Day 0), the C2 subpopulation expanded markedly and was enriched in the G2/M phase of the cell cycle, suggesting a state of active proliferation coinciding with the acute systemic inflammatory response. IGF1R is the cognate receptor for insulin-like growth factor 1 (IGF1), a pleiotropic growth factor with well-established roles in tissue homeostasis, cellular survival, and metabolic regulation [67, 68]. Its expression as a defining marker of an SIRS-expanded NK cell subpopulation indicates that the IGF1 signaling axis may be co-opted during systemic inflammation to modulate NK cell biology. It should be acknowledged that sepsis progression is accompanied by gradual depletion of IGF1 and insulin-like growth factor binding protein-3 (IGFBP3), along with a progressive increase in serum growth hormone (GH) levels [69, 70]. Therefore, the enrichment of the C2 IGF1R + NK cell subpopulation may reflect NK cells attempting to sustain IGF1R-mediated effector programs in the face of falling systemic IGF1 concentrations. In addition, human peripheral blood NK cells actively secrete IGF1 and this endogenous autocrine IGF1 is essential for NK cell cytotoxic function [71]. As SIRS progresses and systemic IGF1 depletion becomes more profound, both the autocrine capacity and paracrine IGF1 availability decline, the C2 IGF1R + NK cell subpopulation contracts, and IGF1R-dependent NK cell effector programs are progressively impaired contributing to the well-documented NK cell dysfunction during the immunosuppressive phase of SIRS. Consistent with previous studies, our results also establish that IGF1R is functionally required for the maintenance of basal NK cell effector competence and for the full induction of IGF1-driven NK cell activation, providing in vitro experimental validation for the biological significance of the C2 IGF1R + NK cell subpopulation identified in the scRNA-seq analysis of healthy donors with LPS-induced SIRS.

The expansion of C2 IGF1R + NK cells during the acute phase of SIRS has potentially important implications for SIRS pathophysiology and treatment. SIRS is characterized by a biphasic immune response: an initial hyperinflammatory phase followed by an immunosuppressive phase. During the early hyperinflammatory phase, the expansion of IGF1R + NK cells, functionally primed to produce high levels of IFN-γ and TNF-α, may contribute to the cytokine storm and systemic inflammation characteristic of acute SIRS. Conversely, the subsequent decline of C2 IGF1R + NK cells by Day 7 may reflect activation-induced exhaustion or apoptosis, contributing to the immunosuppressive phase. From a therapeutic standpoint, the present data suggest that targeted modulation of IGF1R signaling in NK cells could be a novel strategy to influence the course of SIRS. Dampening IGF1R-mediated NK cell hyperactivation during the acute phase may attenuate excessive systemic inflammation, whereas preserving or restoring IGF1R signaling during the immunosuppressive phase could help sustain NK cell anti-pathogen and anti-viral surveillance functions.

Our study has several limitations. On the one hand, we lacked prognosis-related data and thus could not determine the association between these NK cell subpopulations and clinical prognosis [72]. Given that the timing and course of NK cell therapy are highly dependent on the intrinsic state of NK cells, comprehensive immune monitoring [73] and scoring of disease progression [74] may deepen our understanding of the pathogenesis and promote the optimization of clinical protocols for such immunotherapeutic agents. On the other hand, the sample size included in the present study is relatively limited. Future studies with a larger sample size are warranted. In addition, our current findings are mainly based on bioinformatics analysis of public scRNA-seq datasets, and no in vivo experimental validation has been performed. Complementary experimental studies, such as in vitro NK cell isolation and functional assays [75, 76] (e.g., cytotoxicity assays, cytokine secretion assays) using primary samples and in vivo validation in animal models [77], should be conducted to further confirm the phenotypic and functional alterations of the identified NK cell subpopulations, as well as the molecular mechanisms underlying their dynamic changes during SIRS. Moreover, our work only explored IGF1/IGF1R signaling in NK cells while ignoring its extensive regulatory roles in systemic metabolism and other key immune cells. Systemic IGF1R targeting therapy may induce unforeseen off-target adverse effects by interfering with physiological homeostasis of these cell populations and whole-body metabolic balance. Subsequent research needs to evaluate these potential side effects and develop cell-specific targeted strategies to reduce non-specific pathway suppression before clinical application for SIRS.

In summary, NK cells are pivotal for pathogen clearance in the early phase of SIRS and also play a critical role in preventing secondary infections or viral reactivation in patients during the immunosuppressive phase. Our study identified 6 NK cell subpopulations with distinct transcriptional identities, dynamic composition, and functional states across the course of SIRS. Among these, the C2 IGF1R + NK cell subpopulation emerged as a key population that expanded specifically during early SIRS, and in vitro functional experiments demonstrated that IGF1/IGF1R signaling positively regulates NK cell inflammatory activation, cytotoxic effector function, and cellular survival, establishing a mechanistic basis for the biological significance of this subpopulation. Although circulating IGF1 levels decline steadily in sepsis owing to growth hormone resistance [69, 70], our data, alongside NK cell autocrine IGF1 production and the parallel temporal changes of the C2 subpopulation and IGF1 axis, support that IGF1/IGF1R signaling regulates NK cell responses across SIRS progression. Based on these findings, defects and dysfunction of NK cells in SIRS can be reversed through multiple strategies, including NK cell adoptive transfer [78, 79], blockade of inhibitory receptors [80–83], and treatment with NK cell-activating cytokines such as IL-12 [84], IL-15, and IL-18. Furthermore, the IGF1/IGF1R axis represents a newly identified regulatory pathway that could be exploited to enhance or dampen NK cell activity at different stages of SIRS. However, during the acute phase of SIRS, neutralization of specific inflammatory mediators produced by NK cells or direct targeting of distinct NK cell subpopulations, including IGF1R + NK cells, may serve as a key strategy to dampen the systemic inflammatory response.

## Conclusion

NK cells play a central role in maintaining immune homeostasis. The present study performed an in-depth investigation into the heterogeneity and function of NK cells in SIRS. Using single-cell transcriptomic analysis, we identified 6 NK cell subpopulations, among which C0 and C2 NK cells were found to be the dominant subpopulations in the early stage of SIRS. We further characterized the dynamic transition, function, intercellular communication, and TF activity of these subpopulations in detail, with additional validation via in vitro cellular experiments. These findings provide a theoretical basis for identifying novel biomarkers and potential therapeutic targets for SIRS and will facilitate the development of new therapeutic strategies in the future.

## Supplementary Information

Below is the link to the electronic supplementary material.

Supplementary Material 1

## Data Availability

The single-cell data utilized for this systemic inflammation research are publicly available in the GEO database (https://www.ncbi.nlm.nih.gov/geo) under accession number GSE212092.
