# Supplementary material for: Single-cell transcriptomic landscape delineates pathogenic NK cell subpopulations and key mediators in systemic inflammatory response syndrome
Source: Clin Exp Med. 2026 Aug 19;26(1):379. doi: 10.1007/s10238-026-02285-2 (PMC13759699; doi:10.1007/s10238-026-02285-2)
Supplement: Supplementary file 1 [file 10238_2026_2285_MOESM1_ESM.docx]

Supplementary Table 1. siRNA sequences and primer sequences used in this study

| **Category** | **Gene** | **Sequence (5′→3′)** |
| --- | --- | --- |
| siRNA | si-IGF1R-1 (sense) | CAACAGUGGUCAUCAUGGAACUGUTT |
| siRNA | si-IGF1R-1 (antisense) | ACAGUUCCAUGAUGACCACUGUUGTT |
| siRNA | si-IGF1R-2 (sense) | UGACUGUGAAAUCUUCGGCTT |
| siRNA | si-IGF1R-2 (antisense) | GCCGAAGAUUUCACAGUCATT |
| siRNA | si-Control (sense) | UUCUCCGAACGUGUCACGUTT |
| siRNA | si-Control (antisense) | ACGUGACACGUUCGGAGAATT |
| qRT-PCR primer | IGF1R-F | GCTGTGTGAGAAGACCACCA |
| qRT-PCR primer | IGF1R-R | CACATCCAGCAGCTCGTTGT |
| qRT-PCR primer | IFNG-F | ATGTCCAACGCAAAGCAATACAT |
| qRT-PCR primer | IFNG-R | TGGACCTGTGGGTTGTTGAC |
| qRT-PCR primer | TNF-F | CCTCTCTCTAATCAGCCCTCTG |
| qRT-PCR primer | TNF-R | GAGGACCTGGGAGTAGATGAG |
| qRT-PCR primer | IL1B-F | ATGATGGCTTATTACAGTGGCAA |
| qRT-PCR primer | IL1B-R | GTCGGAGATTCGTAGCTGGA |
| qRT-PCR primer | IL6-F | ACTCACCTCTTCAGAACGAATTG |
| qRT-PCR primer | IL6-R | CCATCTTTGGAAGGTTCAGGTTG |
| qRT-PCR primer | GZMB-F | TGGTGGCTTCCTGATACGAG |
| qRT-PCR primer | GZMB-R | GCCATTGTTTCGTCCATAGGAG |
| qRT-PCR primer | PRF1-F | AGGTGGAGGCCCAAACTTAC |
| qRT-PCR primer | PRF1-R | TGAGGTTGAAGTGGGTGCC |
| qRT-PCR primer | NKG7-F | CAGGACCTGAAGGTGGTGAA |
| qRT-PCR primer | NKG7-R | TGGTGATGTCCTGTTCCACA |
| qRT-PCR primer | GNLY-F | CCTGGGAAAGATGCCCTTGT |
| qRT-PCR primer | GNLY-R | GGTCTTTGCGGATCTTGGGT |
| qRT-PCR primer | GAPDH-F | GGAGCGAGATCCCTCCAAAAT |
| qRT-PCR primer | GAPDH-R | GGCTGTTGTCATACTTCTCATGG |
